# Whole brain functional connectivity: Insights from next generation neural mass modelling incorporating electrical synapses

**DOI:** 10.1371/journal.pcbi.1012647

**Published:** 2024-12-05

**Authors:** Michael Forrester, Sammy Petros, Oliver Cattell, Yi Ming Lai, Reuben D. O’Dea, Stamatios Sotiropoulos, Stephen Coombes

**Affiliations:** 1 Centre for Mathematical Medicine and Biology, School of Mathematical Sciences, University of Nottingham, Nottingham, United Kingdom; 2 Faculty of Medicine & Health Sciences, University of Nottingham, Nottingham, United Kingdom; École Normale Supérieure, College de France, CNRS, FRANCE

## Abstract

The ready availability of brain connectome data has both inspired and facilitated the modelling of whole brain activity using networks of phenomenological neural mass models that can incorporate both interaction strength and tract length between brain regions. Recently, a new class of neural mass model has been developed from an exact mean field reduction of a network of spiking cortical cell models with a biophysically realistic model of the chemical synapse. Moreover, this new population dynamics model can naturally incorporate electrical synapses. Here we demonstrate the ability of this new modelling framework, when combined with data from the Human Connectome Project, to generate patterns of functional connectivity (FC) of the type observed in both magnetoencephalography and functional magnetic resonance neuroimaging. Some limited explanatory power is obtained via an eigenmode description of frequency-specific FC patterns, obtained via a linear stability analysis of the network steady state in the neigbourhood of a Hopf bifurcation. However, direct numerical simulations show that empirical data is more faithfully recapitulated in the nonlinear regime, and exposes a key role of gap junction coupling strength in generating empirically-observed neural activity, and associated FC patterns and their evolution. Thereby, we emphasise the importance of maintaining known links with biological reality when developing multi-scale models of brain dynamics. As a tool for the study of dynamic whole brain models of the type presented here we further provide a suite of C++ codes for the efficient, and user friendly, simulation of neural mass networks with multiple delayed interactions.

## Author summary

The neuroimaging of functional connectivity (FC) is a highly useful proxy for brain dynamics in both health and disease. Nonetheless, the investigation of the mechanistic origins of FC is still ongoing. The combination of connectome data with realistic, yet computationally cheap, biophysically realistic models of neural activity is a powerful tool to complement the advances being made in e.g., diffusion and functional magnetic resonance imaging, electro- and magneto-encephalography, tractography, and inference. To date this modelling approach has mostly relied on biologically motivated, yet phenomenological, networks of inter-connected neural masses, with only simple representations of chemical synapses and little if any attention paid to the contribution of electrical synapses to large scale brain dynamics. Here, we redress this deficiency with the study of a new class of neural mass models for cortical activity that are derived from an underlying microscopic model of spiking neurons with interactions mediated by both chemical and electrical synapses. At the whole brain level we show how the combination of regional dynamics, synaptic processing, and long range axonal delayed interactions can contribute to patterns of FC that are best fit to real data with the inclusion of gap junctions within the new modelling framework.

## Dedication

We would like to dedicate this paper to the memory of our dear friend and colleague, Yi Ming Lai. Although beginning with us on the journey to write this paper sadly he did not end that journey with us. RIP Yi Ming Lai 1988–2022.

## Introduction

Since the mid 1990s functional connectivity (FC) has been recognised as a practical tool to characterise the patterns of correlation and coherence in neural activity between brain regions based on temporal similarity [1], especially as measured with neuroimaging modalities such as functional magnetic resonance imaging (fMRI) during the resting state. Changes in FC are believed to reflect higher brain functions [2–4] and have been extensively studied in the context of changes in cognitive processing during aging [5, 6] and due to neurological disease [7–9]. FC patterns can evolve over tens of seconds, with essentially discontinuous shifts from one short term state to another [10]; however, the maintenance of even the relatively short term static patterns of FC is still relatively poorly understood from a mechanistic perspective. This is despite the widespread use of FC in distinguishing between healthy and pathological brain states [11]. Computational modelling has proven an invaluable tool for gaining insight into the potential mechanisms that can give rise to whole-brain network dynamics, including FC. Activity in this area of computational neuroscience and neuroinformatics is exemplified by that of the Virtual Brain project that combines connectome data, such as that available from the Human Connectome Project (HCP) [12], with *neural mass* modelling and can map onto a wide range of neuroimaging modalities [13]. By neural mass we mean the population models of neural activity of the type introduced in the 1970s by Wilson and Cowan [14] and refined subsequently by Zetterberg *et al*. to better fit electroencephalography recordings [15], and being more widely popularised by the work of Jansen and Rit [16]. Such models are biologically-inspired but essentially phenomenological descriptions, with state variables that track coarse-grained notions of the average membrane potential or population firing rate. They are expected to provide appropriate levels of description for many thousands of near identical interconnected neurons with a preference to operate in synchrony. This latter assumption is especially important for the generation of a sufficiently strong physiological signal that can be detected by non-invasive neuroimaging. This neural mass approach has a benefit for large scale whole brain models since each brain region can be represented by a relatively small number of differential equations in simulation studies. However, the downside is a potential disconnect from biophysical reality since such models make no attempt to describe the evolution of individual neurons within a population, nor attempt to give an account of realistic descriptions of synaptic currents that depend on specific excitatory and inhibitory synaptic reversal potentials; as such, model parameter choices and the dynamics they underlie pose problems for clear biological interpretability, with perhaps the sole exception being the work of Liley *et al*. [17]. Of course, one recourse is to use detailed simulations of large numbers of cells in the spirit of the Human Brain Project [18], though it is often hard to gain insight about operating mechanisms from such studies. Rather, a bridge from microscopic to macroscopic levels of brain activity is desired to aid in the understanding of the mechanism of brain dynamics. Although we do not yet have a general statistical neurodynamics theory to provide this, some recent progress has been made for a specific choice of network of so-called *θ*-neurons [19, 20]. This model is formally equivalent to the voltage based quadratic integrate-and-fire (QIF) neuron that also admits to a mean-field reduction [21, 22]. Importantly, the inclusion of realistic chemical and electrical synapses is possible within these networks prior to reduction, and gives rise to a mean-field model that takes a similar form to a standard neural mass model, with an additional dynamical equation to describe the evolution of within-population synchrony and whose parameters are explicitly linked to the underlying neural biophysics [23]. Interestingly, the sigmoidal firing rate function so ubiquitous in phenomenological neural mass modelling is superseded by a firing rate *f*(*Z*) ∝ Re((1 − Z*)/(1 + Z*)), where Z∈C denotes the Kuramoto order parameter, so that population firing is intimately linked to the degree of within population synchrony. A corresponding dynamical equation for the evolution of *Z* couples back to the models for chemical and electrical synaptic currents. This *derived* low dimensional neural mass model has a far richer dynamical repertoire than standard neural mass models [21–30], and maintains a strong link to single cell and synapse dynamics that makes it ideally suited for large scale brain modelling. As such we refer to it as a *next generation* neural mass model; see [31] for a recent perspective and note that the Virtual Brain project has very recently incorporated such more realistic neural mass models [32].

**Fig 1:**
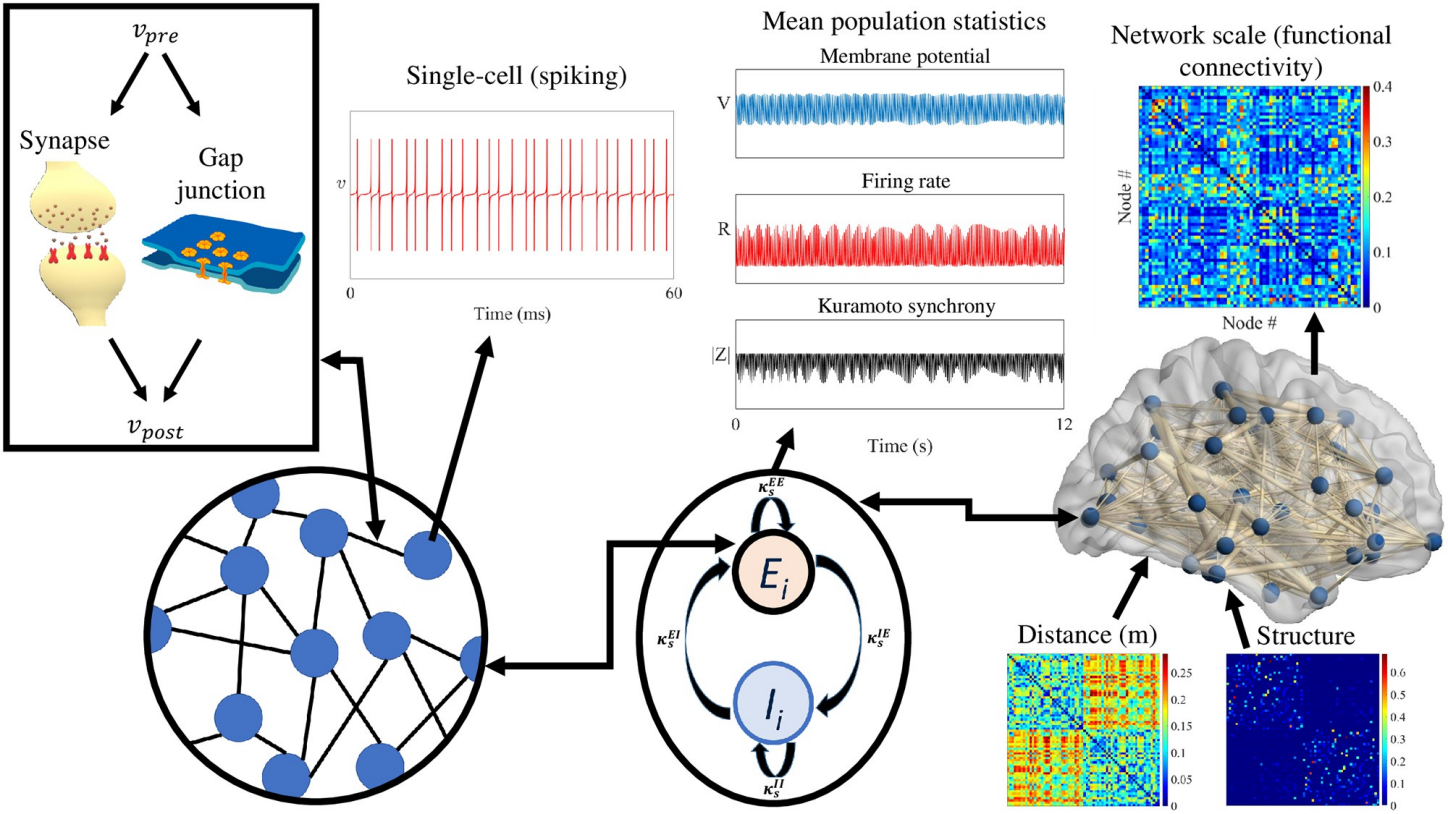
A schematic overview of the model used in this work. From left to right, the underlying components of the model are presented at increasing spatial scales. On the far left is a network of spiking neurons, with interactions between pre- (*v*_*pre*_) and post-synaptic (*v*_*post*_) cells. There are two forms of neural interaction: *gap-junctions* (modelled with a fixed resistance *κ*_*v*_, which we refer to as gap-junction coupling strength) and *synapses* (modelled as a variable resistor with conductivity *g* with a synaptic reversal potential *v*_*syn*_). Via a reduction methodology that invokes the Ott-Antonsen ansatz [39], a low-dimensional description of the spiking neurons’ activity can be formulated, in the infinite-neuron limit, allowing for a computationally tractable model of large-scale neural populations. For a macroscopic region of the cortex, we consider two such populations, one excitatory and one inhibitory, that have both reciprocal and self-coupled synapses and gap-junctions. Using the reduced model, we run forward simulations of average, or mean-field, population statistics: membrane potential, firing rate and Kuramoto order parameter of synchrony (see Eq (6)). Each E–I pair is embedded in a whole-brain network of 68 cortical regions and we allow long-range synaptic connections between excitatory populations, with connectivity strengths and conduction delays derived from white-matter diffusion MRI data. Network behaviour is quantified using pairwise-correlation analogous to methods used to compute functional connectivity from empirical MEG/EEG time series.

In this paper we focus on the description and use of a next generation neural mass model in a network built with human connectome data to determine how FC emerges as a function of physiologically meaningful model parameters that relate, for example, to local excitability, synaptic time-scale, degree of axonal myelination, and strength of gap-junction coupling. This is a major step that moves beyond previous use of phenomenological neural mass models, as in [33–35], to address the *structure-function* question of large scale neuroimaging [36–38]. Fig 1 provides a schematic overview of the neural mass model and its underlying spiking dynamics; through the incorporation of structural connectivity (SC) data we exploit this model to simulate large-scale cortical dynamics and thereby to study emergent FC patterns.

To study in detail the relationship between neural mass dynamics, evoked functional connectivity and the underlying connectome, in the first instance we employ a proxy FC constructed from the eigenstructure of the anatomical connectivity matrix (corresponding to human connectome data) to explore those patterns that such connectivity naturally supports. Subsequently, we employ a linear stability analysis to incorporate interaction between connectivity structure and model dynamics in determining evoked functional patterns that are acquired in fMRI and magnetoencephalography (MEG). Although some explanatory power of the model is established through these analyses, direct numerical simulations of the full nonlinear model away from bifurcation are found to have much better correspondence with empirical data. We further show that the strength of gap junction coupling has a strong effect on emergent network dynamics, and is a tunable parameter that allows for improved fits of model FC to empirical FC. Additionally we highlight that previous modelling work has relied, in part, on the use of noise [40] or fixed offset delays [41] to generate fluctuating time-series capable of generating synthetic FC patterns; the model presented here is sufficiently rich to require neither.

Complementary to the modelling and theoretical work presented here we have produced a freely available suite of C++ tools for the efficient simulation of neural mass network models, with conduction delays between brain regions, where the user can specify the choice of structural connectivity and path length data for axonal fibre tracts.

## Methods

### Network model of cortical activity

Here we describe the new type of neural mass model considered as a model for localised cortical activity and its subsequent use as a node in a larger whole brain network. The mean field model can be derived from a network of quadratic integrate and fire (QIF) neurons, with a single cell model in isolation able to replicate many of the properties of a real cortical cell, including a low firing rate. Importantly the spiking network model can incorporate event driven chemical synaptic interactions as well as direct electrical connections. For a recent discussion of the derivation of the mean field model see [23].

Synaptic currents are modelled using conductance changes and have the form *κ*_*s*_*g*(*t* − *T*)(*v*_*syn*_ − *v*(*t*)), where *κ*_*s*_ represents the strength of chemical synaptic coupling, *g* represents a time-dependent conductance change triggered by the arrival of an action potential at time *T*, *v*(*t*) is the cell membrane voltage at time *t*, and *v*_*syn*_ is the reversal potential of the synapse. This conductance response *g*(*t*) will be taken to be the Green’s function of a linear differential operator *Q*, so that *Qg* = *δ* where *δ* is a delta-Dirac spike. Throughout the rest of this paper we shall take *g*(*t*) to be an *α*-function, so that *g*(*t*) = *α*^2^*t* exp(−*αt*)*H*(*t*), where *H* is a Heaviside step function. In this case the operator *Q* is second order in time and given by

$$\begin{array}{c} Q=(1+\frac{1}{\alpha }\frac{\text{d}}{\text{d}t}{)}^{2}, \end{array}$$ (1)

where *α*^−1^ is the time-to-peak of the synapse. When compared to the linear integrate-and-fire model the QIF model has a representative spike-shape though not one as realistic as that of a true action potential [26, 42]. The QIF model can communicate both sub-threshold and spiking voltages via direct electrical synapses. In contrast to the previously described chemical synapses, an electrical synapse is an electrically conductive link between two adjacent nerve cells that is formed at a fine gap between the pre- and post-synaptic cells, permitting a direct electrical connection between them. It is common to view these so-called gap junctions as a channel that conducts current according to a simple ohmic model. For two neurons with voltages *v*_*n*_ and *v*_*p*_ the current flowing into cell *n* from cell *p* is *κ*_*v*_(*v*_*p*_ − *v*_*n*_), where *κ*_*v*_ represents the strength of gap junction coupling. This gives rise to a state-dependent interaction. A large globally coupled network of interacting QIF neurons, with some heterogeneity determined by a set of non-identical background drives to each cell, has a set of network equations that can be written in the form

$$\begin{array}{c} \tau {\dot{v}}_{n}=\eta_{n}+v_{n}^{2}+\kappa_{s}g\left(v_{\text{syn}}-v_{n}\right)+\frac{\kappa_{v}}{P}\sum_{p=1}^{P}\left(v_{p}-v_{n}\right),\;n=1,\ldots ,P, \end{array}$$ (2)

where the voltage is reset to *v*_r_ → −∞ whenever *v*_th_ → ∞. Here, *η*_*n*_ is a random variable drawn from a Cauchy distribution with center *η*_0_ and width at half maximum Δ, and *τ* is the cell membrane time-constant. We will work in the thermodynamic limit (*P* → ∞), and choose a model of global conductance change that is driven by delta–Dirac spikes in the form:

$$\begin{array}{c} Qg=\underset{P\to \infty }{\text{lim}}\frac{1}{P}\sum_{p=1}^{P}\sum_{m\in Z}\delta \left(t-T_{p}^{m}\right), \end{array}$$ (3)

where *Q* is given by (1), and $T_{p}^{m}$ denotes the *m*th time that neuron *p* spikes (defined by the time that the neuronal voltage reaches threshold). This large system of interacting QIF neurons, defining the microscopic dynamics, is illustrated in the left hand subplot of Fig 1. A large globally coupled network of interacting QIF neurons, with some heterogeneity determined by a set of non-identical background drives to each cell, then admits to the low dimensional mean field description *Qg* = *κ*_*s*_*R*, with

$$\begin{array}{c} \tau \dot{R}=-R\left(g+\kappa_{v}\right)+2RV+\frac{\Delta }{\pi \tau }, \end{array}$$ (4)

$$\begin{array}{c} \tau \dot{V}=\eta_{0}+V^{2}-\pi^{2}\tau^{2}R^{2}+g\left(v_{syn}-V\right). \end{array}$$ (5)

Here, the dynamical variables *R* and *V* represent the instantaneous mean firing rate (the fraction of neurons firing at time *t*) and the average membrane potential. Note that there is an alternative interpretation of the parameters *η*_0_ and Δ, when the QIF neurons are homogeneous but subject to independent, identically distributed Cauchy white noise with center *η*_0_ and width Δ [43–45]. Interestingly, the pair (*R*, *V*) can be related to the Kuramoto order parameter for synchrony by a Möbius transformation. At the single neuron level the QIF model can be transformed to a circular *θ*-neuron model by the half-angle transform *v*_*n*_ = tan(*θ*_*n*_/2). From these individual single neuron *angles* one may naturally define a measure of synchrony in terms of the complex number *Z* defined by:

$$\begin{array}{c} Z=\underset{P\to \infty }{\text{lim}}\frac{1}{P}\sum_{p=1}^{P}{\text{e}}^{\text{i}\theta_{p}}. \end{array}$$ (6)

This is the so-called Kuramoto order parameter. In a complex polar representation with *Z* = |*Z*|e^iΘ^, the magnitude |*Z*| provides a measure of population synchrony whilst Θ ∈ [0, 2*π*) defines an angle. The complex variable *W* = *πτR* + i*V* can be related to Kuramoto order parameter *Z* according to the conformal map *Z* = (1 − W*)/(1 + W*), where W* is the complex conjugate of *W* [21], and see [31] for a recent discussion about the origins of this Möbius transformation. From this it can be seen that the firing rate of the population can be constructed as function of *Z* according to *R* = (*πτ*)^−1^Re((1 − Z*)/(1 + Z*)).

The extension of (4) and (5) to describe interacting excitatory and inhibitory sub-populations, with both reciprocal and self connections, is straightforward with the introduction of indices *E* and *I*, and of four distinct synaptic reversal potentials with $v_{syn}^{a,E}>0$ and $v_{syn}^{a,I}<0$ for *a* ∈ {*E*, *I*}. This gives rise to *M* = 12 first order differential equations of an excitatory-inhibitory population in the form

$$\begin{array}{c} \tau_{a}{\dot{R}}_{a}=-R_{a}\sum_{b}\left(g_{ab}+\kappa_{v}^{ab}\right)+2R_{a}V_{a}+\frac{\Delta_{a}}{\pi \tau_{a}}, \end{array}$$ (7)

$$\begin{array}{c} \tau_{a}{\dot{V}}_{a}=\eta_{0}^{a}+V_{a}^{2}-\pi^{2}\tau_{a}^{2}R_{a}^{2}+\sum_{b}g_{ab}\left(v_{syn}^{ab}-V_{a}\right)+\sum_{b}\kappa_{v}^{ab}\left(V_{b}-V_{a}\right), \end{array}$$ (8)

$$\begin{array}{c} {\dot{g}}_{ab}=\alpha_{ab}\left(s_{ab}-g_{ab}\right),\;{\dot{s}}_{ab}=\alpha_{ab}\left(\kappa_{s}^{ab}R_{b}-s_{ab}\right). \end{array}$$ (9)

We can regard Eqs (7), (8) and (9) as a mesoscopic description of population activity as illustrated in the middle subplot of Fig 1. This model may then be employed to study the dynamics of networks of neural masses by treating a network of *N* such nodes, each built from an (*E*, *I*) pair, and identified with a further index *i* = 1, …, *N*, where *N* is the total number of nodes in the network. Interactions between nodes *i* and *j* are then determined by a SC matrix (such as that obtained from brain connectome data) with components *w*_*ij*_ ≥ 0, assuming that node-node interactions only occur via excitatory pathways. Giving further consideration to the axonal delays that arise when signals are communicated between different brain regions, described by a set of delays *T*_*ij*_, we arrive at the full network equations given by (7)–(9) with the inclusion of a term $\sum_{j=1}^{N}g_{ij}\left(t\right)\left(v_{syn}^{ij}-V_{E_{i}}\right)$ in the right hand side of the equation for ${\dot{V}}_{E}$ (8) and a term $-R_{E}\sum_{j=1}^{N}g_{ij}$ in the right hand side of ${\dot{R}}_{E}$ (7) at each node *i*, where $V_{E_{i}}$ is the average excitatory membrane potential in the *i*^th^ node, and

$$\begin{array}{c} Q_{ij}g_{ij}\left(t\right)=k_{\text{ext}}w_{ij}R_{E_{j}}\left(t-T_{ij}\right), \end{array}$$ (10)

wherein $R_{E_{j}}$ is the firing rate of the excitatory population in the *j*^th^ node. Here, *Q*_*ij*_ is the differential operator ${\left(1+\alpha_{ij}^{-1}\text{d}/\text{d}t\right)}^{2}$ with Green’s function $\eta_{ij}\left(t\right)=\alpha_{ij}^{2}t{\text{e}}^{-\alpha_{ij}t}H\left(t\right)$, $v_{syn}^{ij}$ represents the (excitatory) reversal potential of the synapse between nodes *i* and *j*, and *k*_ext_ is a global coupling strength. Hence, the full network is described by (*M* + 2*N*)*N* delay differential equations. In this way we arrive at a macroscopic description of large scale brain activity, as illustrated in the right subplot of Fig 1. In practice, the connectivity strengths *w*_*ij*_ and conduction time-delays *T*_*ij*_ are obtained from Human Connectome data, and see section **Structural connectivity and path-length data**. The latter are computed from axonal distances between nodes *i* and *j*, denoted by *d*_*ij*_ as *T*_*ij*_ = *d*_*ij*_/*v* for a fixed uniform action potential conduction speed *v*. In reality this might be better chosen from a *γ*-distribution [46]. Motivated by studies of myelinated axons from the human corpus callosum reported in [47] we have take a representative value of *v* = 12 ms^−1^.

Unless otherwise stated, other parameter values are (with time in seconds and potential in mV): mean population inputs $\eta_{0}^{I}=3.0$, $\eta_{0}^{E}=-2.5$; input distributions’ widths at half maximum Δ_*I*_ = Δ_*E*_ = 0.5; membrane timescales *τ*_*I*_ = 0.012, *τ*_*E*_ = 0.011; synaptic rates *α*_*EE*_ = 50, *α*_*EI*_ = 40, *α*_*IE*_ = 50, *α*_*II*_ = 40, *α*_*ij*_ = 40; synaptic coupling strengths $\kappa_{s}^{EE}=0.5$, $\kappa_{s}^{EI}=0.3$, $\kappa_{s}^{IE}=0.7$, $\kappa_{s}^{II}=0.3$; synaptic reversal potentials $v_{syn}^{aE}=10$, $v_{syn}^{aI}=-10$, $v_{syn}^{ij}=10$; gap-junction coupling strengths $\kappa_{v}^{EE}=0.01$, $\kappa_{v}^{EI}=\kappa_{v}^{IE}=0$, $\kappa_{v}^{II}=0.025$ and network coupling strength *k*_ext_ = 0.2. These default parameters are chosen to be physiologically plausible and such that the network steady state is close to a linear instability (see section **Linear stability analysis**). Since gap-junctions are typically found between inhibitory neurons and mostly between the same kind of neuron we have omitted gap-junction cross-coupling by setting *κ*^*EI*^ = 0 = *κ*^*IE*^ and chosen *κ*^*EE*^ < *κ*^*II*^.

The neural dynamics obtained from the above may be used directly in comparisons with empirically-observed MEG, with the local excitatory firing rate time series *R*_*E*_ being the appropriate variable of interest. In the context of Blood Oxygen Level Dependent (BOLD) fMRI, that infers brain activity by detecting changes in neural blood flow, we employ the simple but commonly-used Balloon–Windkessel model to convert simulated neural activity to a suitable measure of haemodynamic response [48, 49]. Full detail is given in section **Functional Connectivity**.

### Computational methodology

The neural mass equations, described in section **Network model of cortical activity**, were integrated numerically by exploiting a recently developed and purpose-built suite of numerical solvers implemented in C++ for simulating neural mass and field problems, NFESOLVE, and for further detail see Supporting information S1 File.

NFESOLVE takes advantage of parallel processing (via the openMP package) together with efficient, sparse data-structure memory storage, and adaptive time-stepping (effected via a third-order Runge-Kutta scheme) that allows for delay differential equation problems to be integrated in an efficient manner. A key part of the efficiency of this suite is in the storage of only those delayed variables that are required and in computing required delayed states that fall between time-steps of past solution states via a third order Hermite interpolant. The size of the history array is dynamic, only ever storing the values necessary to compute the next step in the integration.

The code, along with a selection of example problems, is stored on a GitHub repository and is available to download at https://github.com/UoN-Math-Neuro/NFESOLVE. The example files include functions and parameters describing the neural mass model, which may be edited to suit the particular model of interest to the user. For ease of use, these files use the Armadillo linear algebra library which has a functionality similar to MATLAB.

### Structural and functional connectivity

**Fig 2:**
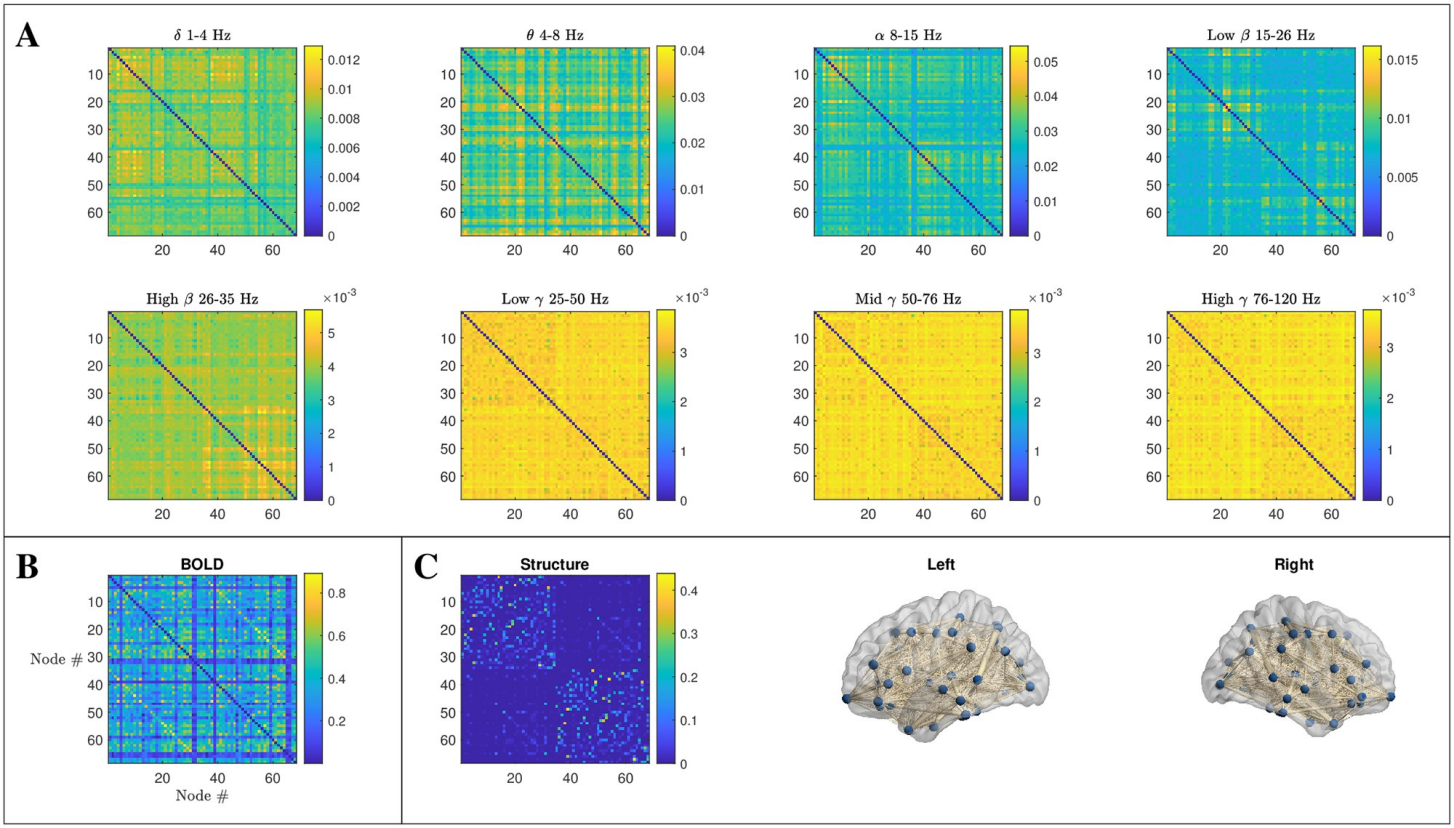
An overview of data types discussed in this paper. In all cases, the data presented is an average over 10 subjects’ datasets from the Human Connectome Project database, and is downsampled onto a 68 node network using the Desikan–Killiany atlas. **A** MEG FC matrices computed via the ‘Multivariate Interaction Measure’ (12) within 8 different bands, which fall into the classical frequency bands *α* to *γ*, where *β* and *γ* are further divided into 2 and 3 sub-bands respectively. **B** BOLD FC is computed by z-scoring parcellated BOLD time series data before computing the pairwise Pearson correlation for all node pairs. **C** Structural data is constructed by applying a probabilistic tractography process to diffusion MRI data. The data is then normalised by row-sum. Visualisations of the structural network are provided for the two hemispheres.

An overview of the structural and functional data that we employ in this study is provided in Fig 2, and summarised in detail below.

#### Structural connectivity and path-length data

The SC was estimated using diffusion MRI data recorded with informed consent from 10 subjects, obtained from the HCP [12]. Briefly, we explain how this data is post-processed to derive connectomic data describing connection strength and path lengths between brain regions, though we direct the reader to [41] and the references therein for a more detailed overview. 60,000 vertices on the white/grey matter boundary surface for each subject [50] were used as seeds for 10,000 tractography streamlines. Streamlines were propagated through voxels with up to three fibre orientations, estimated from distortion-corrected data with a deconvolution model [51, 52], using the FSL package. The number of streamlines intersecting each vertex on the boundary layer was measured and normalised by the total number of valid streamlines. The quotient of path lengths was taken to generate a dense 60,000 node mean distance matrix. The matrix of streamline lengths and the matrix of number of streamlines were symmetrised and further parcellated using the 68-node Desikan–Killiany atlas into regions of interest [53]. The resulting matrices were used to describe connections between brain regions, providing undirected (symmetric), weighted matrices whose elements *w*_*ij*_ and *T*_*ij*_ define the connectivity strengths and conduction time-delays (computed via *d*_*ij*_/*v* for axonal conduction speed, *v* and axonal distance *d*_*ij*_), respectively, of the long-range excitatory connections in equations (10). To generate nodal inputs with commensurate magnitudes, the structural connectivity matrix was normalised by row so that afferent connection strengths for each node sum to unity. This normalisation process permits some of the analysis that we undertake to help explain SC–FC relations (see section **Linear stability analysis**). Moreover, empirical studies have shown that normalisation reduces the effect of confounds reflecting algorithmic choices and ensures better consistency across subjects [54].

#### Functional connectivity

Functional connectivity can be measured using various techniques, notably BOLD/fMRI and EEG/MEG, each with its strengths and limitations. BOLD/fMRI offers high spatial resolution but low temporal resolution, making it well-suited for identifying the location of functional networks. EEG/MEG, on the other hand, provides high temporal resolution but lower spatial resolution, making it excellent for tracking the timing and sequence of brain activity. The integration of data from both modalities is ideal for generating a more complete understanding of the brain’s functional connectivity. Empirical FC matrices that we employ are computed from data obtained from the HCP [12] in two different modalities: MEG and BOLD. Below, we briefly describe this data (though refer the reader to [12] for full detail), together with the methods by which we construct simulated FC from our computational model for comparison.

#### MEG

MEG FC acquired from the HCP was collected and pre-processed using the framework detailed in [55], though in the following we briefly describe the methods used. MEG data was collected using a whole-head MAGNES 3600 system housed in a magnetically shielded room. To account for signal leakage and external noise, independent component analysis (ICA) [56] was performed iteratively, starting from different initial guesses. For each decomposition, independent components were classified as ‘Brain’ or ‘Noise’ using six parameters derived from a large number of recordings: 3 parameters to quantify correlations between signals, power time-series and spectra, and 3 classifying various types of noise. The final independent component classification was made automatically by selecting the iteration accounting for the highest brain component subspace dimensionality and the lowest residual artifact contamination. The MEG sensor positions were then co-registered with the underlying anatomical space and a source reconstruction algorithm [57] was performed to map signal sources onto the anatomical space.

The HCP pipeline that we exploit uses the Multivariate Interaction Measure (MIM) [58, 59] to construct FC matrices from MEG signals. The MIM is designed to maximize the imaginary part of coherence between vectors that describe the three directional components of the MEG signal at each voxel. In simple terms the MIM can be thought of as a process that compares the MEG time-series for each pair of brain regions by calculating a number that shows how strongly each pair of regions is connected (with higher values indicating stronger connectivity). It does so by computing the cross spectrum (a complex correlation matrix in frequency space) between vector signals at different brain regions and then combining all of the eigenvalues of this matrix to define a synchronisation measure between the two signal sources. This single number (for each pair of brain regions) captures the functional connectivity and can be expressed as a trace over a matrix built from real and imaginary sub-blocks of the cross spectrum. We introduce the metric concisely here, but we refer the reader to [58] for a thorough overview.

The cross spectral density matrix (CSD) from independent component time series is estimated by segmenting the data into 1.0s Hanning windows with 50% overlap and applying the Fast Fourier transform to generate complex frequency-dependent vectors $\text{x}=[{\text{x}}_{A}^{⊺}\left(f\right)\;{\text{x}}_{B}^{⊺}\left(f\right){]}^{⊺}$, where **x**_*A*_ and **x**_*B*_ denote the data from two different brain regions. For cortical node pairs, the CSD has the block matrix form, for each frequency *f*:

$$\begin{array}{c} \text{C}\left(f\right)=\left\langle \text{x}\left(f\right)\text{x}{\left(f\right)}^{†}\right\rangle =(\begin{array}{cc} {\text{C}}_{AA}^{R}\left(f\right)+\text{i}{\text{C}}_{AA}^{I}\left(f\right) & {\text{C}}_{AB}^{R}\left(f\right)+\text{i}{\text{C}}_{AB}^{I}\left(f\right)\\ {\text{C}}_{BA}^{R}\left(f\right)+\text{i}{\text{C}}_{BA}^{I}\left(f\right) & {\text{C}}_{BB}^{R}\left(f\right)+\text{i}{\text{C}}_{BB}^{I}\left(f\right) \end{array}), \end{array}$$ (11)

where † denotes the conjugate transpose and 〈…〉 is the mean over epochs; *R* and *I* superscripts denote real and imaginary parts, respectively. Then the MIM for two channels *A* and *B* is defined as:

$$\begin{array}{c} MIM_{AB}\left(f\right)=\text{Tr}(({\text{C}}_{AA}^{R}\left(f\right){)}^{-1}{\text{C}}_{AB}^{I}\left(f\right)({\text{C}}_{BB}^{R}\left(f\right){)}^{-1}({\text{C}}_{AB}^{I}\left(f\right){)}^{⊺}). \end{array}$$ (12)

An analogous metric for cases where each node only has one component, as is the case for the model simulations presented here, is the *Global Interaction Measure* [58] (GIM). In this case, only a single cross-spectrum matrix is needed for each node/channel pair,

$$\begin{array}{c} {\text{C}}_{AB}\left(f\right)=(\begin{array}{cc} c_{AA}\left(f\right) & c_{AB}^{R}\left(f\right)+\text{i}c_{AB}^{I}\left(f\right)\\ c_{AB}^{R}\left(f\right)-\text{i}c_{AB}^{I}\left(f\right) & c_{BB}\left(f\right) \end{array}), \end{array}$$ (13)

where *c*_*AB*_ is the single-component CSD, $\langle {\text{x}}_{A}\left(f\right){\text{x}}_{B}^{*}\left(f\right)\rangle$ and * denotes the complex conjugate. Further, we have,

$$\begin{array}{c} {\left({\text{C}}_{AB}^{R}\left(f\right)\right)}^{-1}=\frac{1}{\text{det}({\text{C}}_{AB}^{R}\left(f\right))}(\begin{array}{cc} c_{BB}\left(f\right) & -c_{AB}^{R}\left(f\right)\\ -c_{AB}^{R}\left(f\right) & c_{AA}\left(f\right) \end{array}). \end{array}$$ (14)

Inserting (13) and (14) into (12), we evaluate the GIM as,

$$\begin{array}{c} GIM_{AB}\left(f\right)=\frac{{\left(c_{AB}^{I}\left(f\right)\right)}^{2}}{c_{AA}\left(f\right)c_{BB}\left(f\right)(1-\frac{{\left(c_{AB}^{R}\left(f\right)\right)}^{2}}{c_{AA}\left(f\right)c_{BB}\left(f\right)})}. \end{array}$$ (15)

For Eqs (12) and (15) the signal is split by band first and then utilised in the formulas.

Simulated FC matrices suitable for comparison with empirical MEG FC are obtained from (15) using local excitatory firing rate time series *R*_*E*_ from Eqs (7)–(10) as the analogue to the MEG signals and FC matrices are constructed using (15).

It is also possible to lever the phase of a Kuramoto order parameter at the level of a model node to construct a pair-wise phase-locking value (PLV) that can also generate a proxy for FC from simulations. If we denote the Kuramoto order parameter at node *j* = 1, …, *N* by $Z_{j}=\left|Z_{j}\right|{\text{e}}^{\text{i}\Theta_{j}}$ then the PLV is a real *N* × *N* matrix with entries

$$\begin{array}{c} R_{ij}=|\underset{t\to \infty }{\text{lim}}\frac{1}{t}\int_{0}^{t}\text{exp}\left(\text{i}\left[\Theta_{i}\left(s\right)-\Theta_{j}\left(s\right)\right]\right)\;\text{d}s|\;. \end{array}$$ (16)

Given that there are two possible Kuramoto order parameters at each node (one for the excitatory population and the other for the inhibitory population) there are two variants of the above; one where the PLV is constructed using only information from excitatory node populations and the other using only inhibitory populations. Other variations are of course possible that mix the two though for simplicity we do not pursue this further. For simplicity we work information from excitatory nodes only.

#### BOLD

Empirical BOLD data was acquired using the steps discussed in [60], which itself uses the HCP minimal pre-processing pipeline as outlined in [50]. Briefly, noise was omitted from BOLD time series using ICA-FIX, designed to remove spatially structured artifactual signals through application of ICA, as well as a machine-learning algorithm constructed to classify independent components into signal or artifact.

The pre-processed resting-state fMRI (rs-fMRI) time series of each subject comprised 4 sessions each spanning 15 min recorded with repetition time of 0.72 s. The rs-fMRI time series was parcellated into 68 areas, then the first 100 time points from each of the BOLD scans were removed to diminish any baseline offsets or signal intensity variation. Time series of each area for each session were z-scored, then all sessions’ time series were concatenated for each subject and z-scored again. The first z-score normalises the individuals recording so that the group level z-score receives an equal contribution for each subject. The FC matrix of each subject was computed using Pearson’s correlation coefficient between the resulting time-series across all real pairs.

We simulate BOLD FC by first passing the model time series though a filter that describes the haemodynamic response to an electrophysiological signal, *S*(*t*), for each node. *S*(*t*) is a component of the system described by (7)–(10), which is chosen to be the excitatory population firing rate *R*_*E*_(*t*) unless otherwise stated. Other choices of signal, such as the average voltage at a node *V*_*E*_(*t*) or the measure of synchrony within an excitatory sub-population |*Z*_*E*_|(*t*) are also natural, though in practice these arbitrary choices appear to make little difference when choosing the filter to be the well-known Balloon–Windkessel model [61]. This is the one that we employ in our treatment of fMRI data. Parameters for a simulated BOLD signal due to a 3T field strength were taken from Appendix A of [62] and other parameters were from [63], as used in a previous computational study of fitting a neural-mass network to fMRI data [60]. The model is described by the system of equations:

$$\begin{array}{cc} \dot{x} & =S\left(t\right)-kx-\gamma \left(f-1\right),\;\dot{f}=x,\\ \tau_{\text{BOLD}}\dot{v} & =f-v^{1/\alpha },\\ \tau_{\text{BOLD}}\dot{q} & =\frac{f}{\rho }\left[1-{\left(1-\rho \right)}^{1/f}\right]-q\left[v^{1/\alpha -1}\right], \end{array}$$ (17)

where *f* and *v* are blood flow and volume, respectively, and *q* is deoxyhaemoglobin content. Parameters (with time in seconds) *ρ* = 0.34, *τ*_BOLD_ = 2, *k* = 0.65, *γ* = 0.41, and *α* = 0.32 are the resting oxygen extraction fraction, haemodynamic transit time, rate of signal decay, rate of flow-dependent elimination, and the Grubb exponent, respectively.

The filter generates an *N*-dimensional solution describing the BOLD signal for each cortical region. The simulated BOLD timeseries, following on from its empirical counterpart, was z-scored before taking the pairwise Pearson correlation, producing an FC matrix.

### In silico *vs*. empirical FC comparison

To interrogate the correspondence between simulated and FC both in empirical and simulated data, and the relationship between anatomical structure and emergent function, we employ the following metrics.

#### Pearson distance

To measure the overlap between simulated and empirical matrices, we employ the Pearson distance,

$$\begin{array}{c} \delta =1-(\text{corr}\left(FC_{\text{sim}},FC_{\text{emp}}\right)-(\left\langle FC_{\text{emp}}\right\rangle -\left\langle FC_{\text{sim}}\right\rangle {)}^{2}), \end{array}$$ (18)

where we have processed the FC data into vectors (*FC*_sim_ and *FC*_emp_) of the elements above the leading diagonals, due to their symmetry. corr(X, Y) is the Pearson correlation between two vectors and 〈⋅〉 is the mean.

#### Structure-function clustering

We employ the recently-developed measure of multiplex structure-function clustering that measures the disparity between weighted anatomical and functional networks [64] (which builds on the unweighted case described in [34]).

For a duplex network comprising a structural layer described by elements $w_{ij}^{\left[1\right]}\in \left[0,1\right]$ and FC layer $w_{ij}^{\left[2\right]}\in \left[0,1\right]$, this clustering measure is defined by

$$\begin{array}{c} C_{\text{wsf}}\left(i\right)=\frac{\sum_{j}\sum_{k,k\neq j}w_{ij}^{\left[1\right]}w_{jk}^{\left[2\right]}w_{ki}^{\left[1\right]}\left(1-w_{jk}^{\left[1\right]}\right)}{\sum_{j}\sum_{k,k\neq j}w_{ij}^{\left[1\right]}w_{ki}^{\left[1\right]}\left(1-w_{jk}^{\left[1\right]}\right)}. \end{array}$$ (19)

The average clustering coefficient across a multiplex network of *N* nodes per layer is obtained via $C_{\text{wsf}}=\left(1/N\right)\sum_{i=1}^{N}C_{\text{wsf}}\left(i\right)$.

### Linear stability analysis

For simplicity we restrict the discussion here to the choice of a connectivity matrix that is row sum normalised, namely $\sum_{j=1}^{N}w_{ij}=1$. This simplifies the construction of the steady state and its linear stability analysis. For a similar reason we make the assumption that the reversal potentials, $v_{syn}^{ij}$, are large in the microscopic dynamics and so input currents from other nodes in the macroscopic network equations are not shunted (*i.e*., do not depend on voltage). Introducing a vector $x_{i}\in R^{M}$ to represent the *M* local variables (*R*_*E*_, *R*_*I*_, *V*_*E*_, *V*_*I*_, *g*_*EE*_, *g*_*EI*_, *g*_*IE*_, *g*_*II*_, *s*_*EE*_, *s*_*EI*_, *s*_*IE*_, *s*_*II*_) for each neural mass, we can write the network Eqs (7)–(10) in the succinct form

$$\begin{array}{c} {\dot{x}}_{i}=f\left(x_{i}\right)+\sum_{j=1}^{N}w_{ij}\eta_{ij}\left(t\right)*\chi \left(x_{j}\left(t-T_{ij}\right)\right),\;i=1,\ldots ,N, \end{array}$$ (20)

where *χ*(*x*) = (0, 0, *R*_*E*_/*τ*_*E*_, 0, 0, 0, 0, 0, 0, 0, 0, 0), $f\in R^{M}$ and * represents temporal convolution (and we absorb a factor of $k_{\text{ext}}v_{syn}^{ij}$ within *w*_*ij*_), namely

$$\begin{array}{c} \eta_{ij}\left(t\right)*\chi \left(x_{j}\left(t-T_{ij}\right)\right)=\int_{-\infty }^{t}\eta_{ij}\left(t-s\right)\chi \left(x_{j}\left(s-T_{ij}\right)\right)\text{d}s. \end{array}$$ (21)

In this case at steady state each node is described by an identical vector with components

$$\begin{array}{c} \bar{x}=\left({\bar{R}}_{E},{\bar{R}}_{I},{\bar{V}}_{E},{\bar{V}}_{I},{\bar{g}}_{EE},{\bar{g}}_{EI},{\bar{g}}_{IE},{\bar{g}}_{II},{\bar{s}}_{EE},{\bar{s}}_{EI},{\bar{s}}_{IE},{\bar{s}}_{II}\right), \end{array}$$ (22)

where ${\bar{g}}_{ab}=\kappa_{s}^{ab}{\bar{R}}_{b}={\bar{s}}_{ab}$ and $\left({\bar{R}}_{a},{\bar{V}}_{a}\right)$ are given by the simultaneous solution of

$$\begin{array}{c} 0=-{\bar{R}}_{a}\sum_{b}\left(\kappa_{s}^{ab}{\bar{R}}_{b}+\kappa_{v}^{ab}\right)+2{\bar{R}}_{a}{\bar{V}}_{a}+\frac{\Delta_{a}}{\pi \tau_{a}}, \end{array}$$ (23)

$$\begin{array}{c} 0=\eta_{0}^{a}+{\bar{V}}_{a}^{2}-\pi^{2}\tau_{a}^{2}{\bar{R}}_{a}^{2}+\sum_{b}\kappa_{s}^{ab}{\bar{R}}_{b}\left(v_{syn}^{ab}-{\bar{V}}_{a}\right)+\sum_{b}\kappa_{v}^{ab}\left({\bar{V}}_{b}-{\bar{V}}_{a}\right)+\delta_{a,E}{\bar{R}}_{a}. \end{array}$$ (24)

Linearising the network equations around the steady state with $x_{i}\left(t\right)=\bar{x}+{\text{e}}^{\lambda t}u_{i}$ for λ∈C and |*u*_*i*_| ≪ 1 gives

$$\begin{array}{c} \lambda u_{i}=\text{D}f\left(\bar{x}\right)u_{i}+\sum_{j=1}^{N}w_{ij}{\tilde{\eta }}_{ij}\left(\lambda \right){\text{e}}^{-\lambda T_{ij}}\text{D}\chi \left(\bar{x}\right)u_{j}, \end{array}$$ (25)

where $\tilde{\eta }\left(\lambda \right)=\int_{0}^{\infty }\text{d}t\;\eta \left(t\right){\text{e}}^{-\lambda t}$ denotes the Laplace transform of *η*, and D*f* and D*χ* are the Jacobians of *f* and *χ* respectively. We have explicitly that ${\tilde{\eta }}_{ij}={\left(1+\lambda /\alpha_{ij}\right)}^{-2}$. Introducing the matrix $\tilde{w}\left(\lambda \right)$ with components $\tilde{w}{\left(\lambda \right)}_{ij}=w_{ij}{\tilde{\eta }}_{ij}\left(\lambda \right){\text{e}}^{-\lambda T_{ij}}$ allows us to rewrite (25) in tensor notation as

$$\begin{array}{c} {}[I_{N}⊗\text{D}f\left(\bar{x}\right)+\tilde{w}\left(\lambda \right)⊗\text{D}\chi \left(\bar{x}\right)-\lambda I_{N}⊗I_{M}]U=0, \end{array}$$ (26)

where *U* = (*u*_1_, …, *u*_*N*_). For want of a better phrase we shall refer to the matrix $I_{N}⊗\text{D}f\left(\bar{x}\right)+\tilde{w}\left(\lambda \right)⊗\text{D}\chi \left(\bar{x}\right)$ as the *network Jacobian*. By introducing a new variable *Y* according to the linear transformation *Y* = (*P* ⊗ *I*_*M*_)^−1^*U*, where *P* is the matrix of normalised right eigenvectors of $\tilde{w}$, we obtain a block diagonal system for *Y*. This is in the form of (26) under the replacement $\tilde{w}\left(\lambda \right)\to \text{diag}\left(\gamma_{1}\left(\lambda \right),\gamma_{2}\left(\lambda \right),\ldots ,\gamma_{N}\left(\lambda \right)\right)$, where *γ*_*μ*_ are the eigenvalues of $\tilde{w}\left(\lambda \right)$. These can be expressed as $\gamma_{\mu }\left(\lambda \right)=\sum_{i,j=1}^{N}\tilde{w}{\left(\lambda \right)}_{ij}u_{i}^{\mu }v_{j}^{\mu }$, where *v*^*μ*^ and *u*^*μ*^ are the right and left eigenvectors respectively of the SC matrix *w*. Thus, the eigenvalues of the linearised network system are given by the set of spectral equations

$$\begin{array}{c} \text{det}[\lambda I_{m}-\text{D}f\left(\bar{x}\right)-\gamma_{\mu }\left(\lambda \right)\text{D}\chi \left(\bar{x}\right)]=0,\;\mu =1,\ldots ,N. \end{array}$$ (27)

The network steady state is stable if Re λ < 0 for all *μ*. Should this stability condition be violated for some value of *μ* = *μ*_*c*_ then we would expect the excitation of the structural eigenmode $v^{\mu_{c}}$. This linear stability analysis is useful for determining points of instability that lead to network oscillations that we employ in the direct simulations. These network oscillations can take the form of synchronised bulk oscillations in which all nodes follow a common periodic orbit with a common frequency, phase-locked patterns in which all nodes follow a common periodic orbit though with a constant phase-shift with respect to one another (with a spatial network pattern predicted by the excited eigenmode close to the bifurcation point), or more exotic behaviours, including travelling waves, expected far from the point of instability. Interestingly, even in the absence of delays, the Jacobian of a linearized whole-brain network model (built from linear coupled phenomenological Epileptor nodes) has been shown to be useful in predicting the properties of traveling epileptic activity [65].

Here we note that Hopf bifurcations, arising when a complex eigenvalue with a non-zero imaginary component crosses the imaginary axis into the right hand complex plane, can be induced even in the absence of delays. However, the presence of delays allows for the excitation of eigenmodes of *w* in a different order to that in the absence of delays [41]. Moreover, many computational studies of coupled oscillators have illustrated that transmission time delays can play a major role in organising network behaviour, see e.g., [66, 67]. The mathematical treatment of the patterns of phase-locked oscillatory network states that can emerge beyond bifurcation is a mathematically challenging one. Nonetheless, for weakly coupled oscillators with delays this can be addressed relatively simply using phase reduction, as recently done by Petkoski and Jirsa [68]. They use this approach to derive a *normalization of the connectome* that can explain the emergence of frequency-specific network cores (including the visual and default mode networks). Moving beyond the limitations of phase reduction to describe networks of interacting delayed limit cycle oscillators one might further envisage the use of recently developed *phase-amplitude* reductions [69, 70], or the use of exact approaches for delayed networks of Amari neural masses [71].

## Results

### Eigenmode analysis

#### Structural eigenmodes

**Fig 3:**
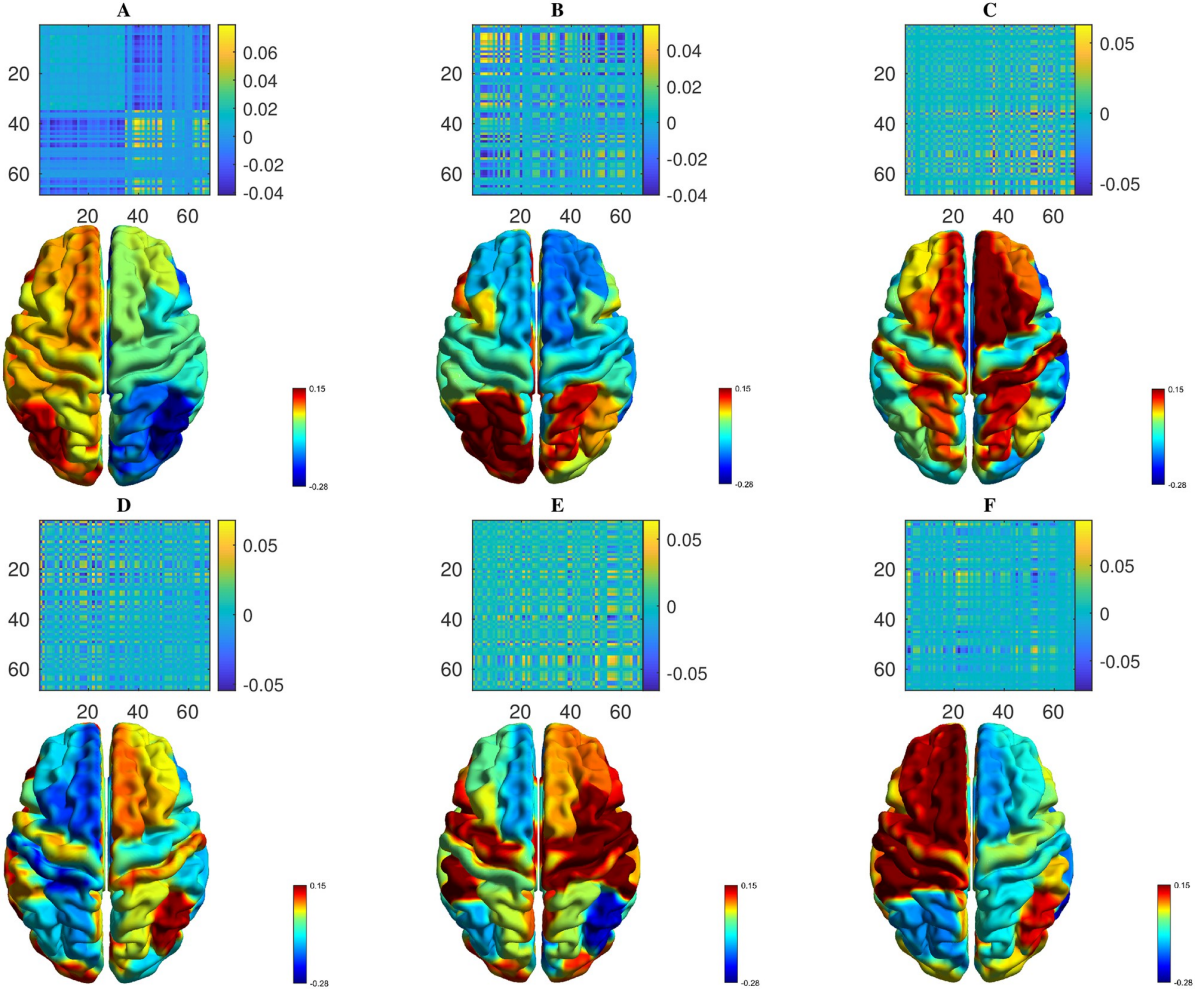
Exemplar proxy FC computed from eigenvectors of the structural connectivity matrix *w*_*ij*_, computed by taking the outer product of an eigenvector with itself. Panels (**A**-**F**) show FC matrices constructed in this way from structural eigenmodes that correspond to the largest six eigenvalues, ordered by decreasing size. Visualisations on a cortical surface are coloured according to the value of normalised eigenvector components, with warmer colours indicating higher values.

Following the linear arguments above, in section **Linear stability analysis**, a starting point to investigate the FC patterns naturally supported by the underlying structure is to compute eigenvectors of the structural connectivity matrix *w*_*ij*_, and construct an FC proxy by the outer product of an eigenvector with itself. Emergent FC can potentially be expected to be formed from a combination of such underlying structural eigenmodes [41]. Fig 3 shows representations of networks constructed in this way, employing each of the first 6 leading eigenvectors (ordered by decreasing size of the corresponding eigenvalue).

**Fig 4:**
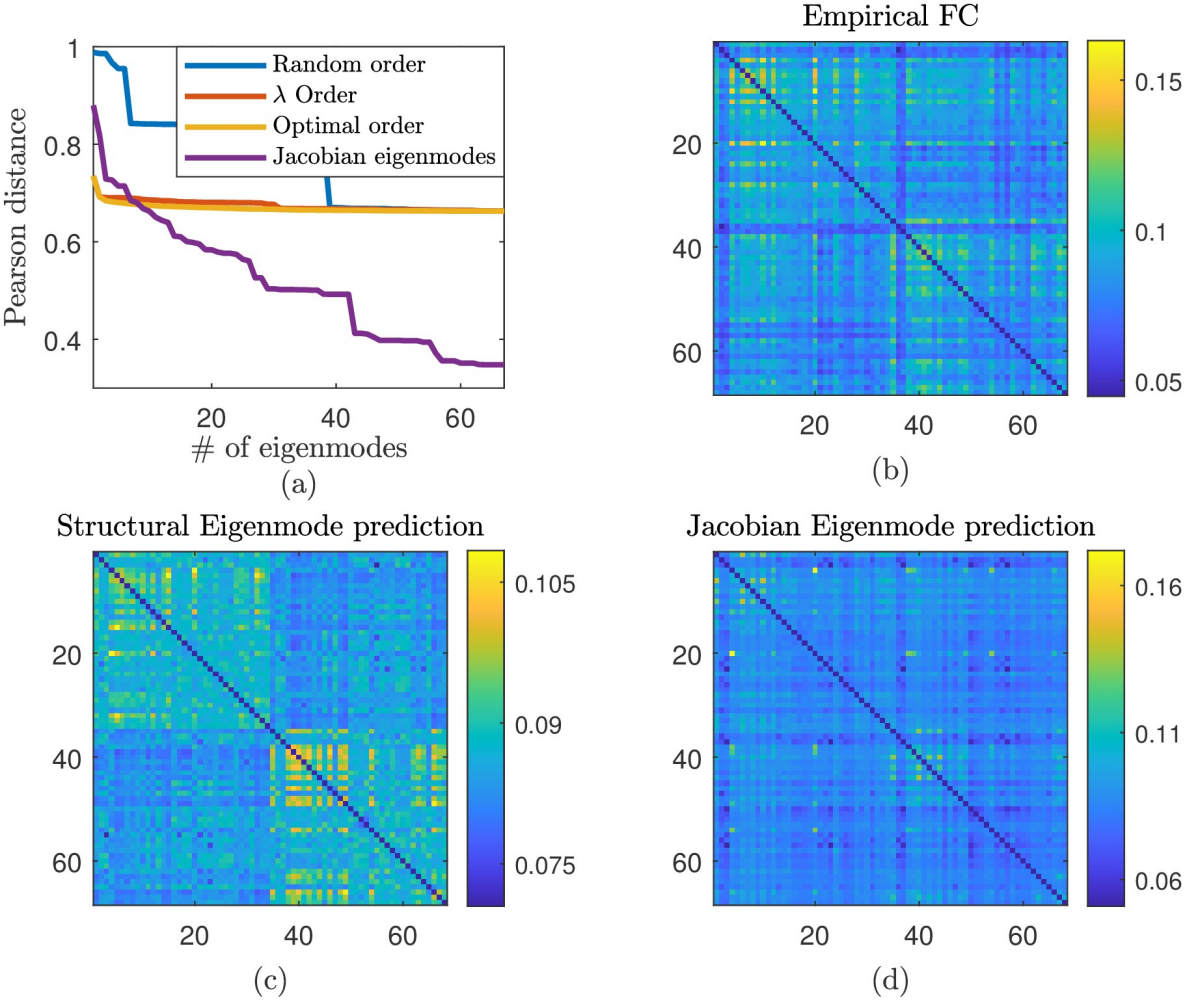
Proxy FC provides limited explanatory power in understanding empirical FC patterns. (a) Accuracy of proxy FC constructed from outer products of eigenvectors of the structural connectivity matrix, or of the network Jacobian, in a similar manner to those presented in Fig 3. Accuracy is measured by Pearson distance from MEG FC obtained from *α*-band activity. FC proxies are computed via iterative linear combination of increasingly many eigenmode FC patterns, with accuracy measured after each subsequent addition; specifically: structural eigenvectors added at random (blue), according to the decreasing size of the corresponding eigenvalue (red), in an order chosen for which the step-wise decrease in error is maximised (orange); and eigenmodes of the network Jacobian, in order of decreasing size of the corresponding eigenvalue (purple). Panels (b–d) provide visual comparison of the most accurate FC proxies obtained from structural eigenmodes and network Jacobian eigenmodes with empirical FC.

Predictions based only on structural information would only be expected to have limited explanatory power, neglecting as they do the influence of local neural dynamical states on emergent FC pattern [35]. To illustrate this, Fig 4 compares the accuracy as measured by Pearson distance from MEG FC obtained from *α*-band activity (see section **Functional connectivity—MEG**) of FC proxies constructed from outer products of eigenvectors of the structural connectivity matrix, or of a reduced network Jacobian, in a similar manner to those presented in Fig 3. Regarding the latter, we compute the eigenvectors of the *NM* × *NM* network Jacobian defined in section **Linear stability analysis** and then construct a projection to $R^{N}$ by considering only the elements corresponding to *R*_*E*_.

Specifically, proxy FC is constructed by linear combination (optimising on the coefficients via MATLAB’s nonlinfit function) of a subset of eigenmodes of either the SC or the Network Jacobian. The order of their addition to the composite FC proxy is as follows: (i) structural eigenmodes, added uniformly at random; (ii) structural eigenmodes, added according to the decreasing size of their corresponding eigenvalue; (iii) structural eigenmodes, in an order chosen for which the step-wise decrease in error between proxy FC and MEG FC is maximised; and (iv) eigenmodes of the network Jacobian (using only the component corresponding to *R*_*E*_), in order of decreasing size of the corresponding eigenvalue. Fig 4(a) shows the incremental improvement in accuracy in comparison to empirical FC, while panels (b–d) compare the example FC proxy matrices resulting from structural or Jacobian eigenmodes (process (ii) and (iv) described previously, in the case of all eigenmodes employed) with empirical data.

Structural information alone is limited in its ability to explain observed FC data, with significant disparity between proxy and empirical FC (Fig 4(a), orange line). In contrast, employing eigenmodes obtained from the network Jacobian, obtained via a linear analysis as described above and hence embedding both structural and dynamical features, provides significant additional improvement. These results highlight how a linear analysis of the dynamical model provides additional explanatory power in understanding empirical FC patterns, in comparison to employing connectomic information alone, but also indicates that the prediction obtained is nonetheless limited in its fidelity.

### Numerical exploration of nonlinear network model

The preceding results highlight the limitations of employing structural data and/or linear analysis in predicting empirical FC. Here we consider the rich detail in neural activity and associated FC patterns supported by the network model (7)–(10) (together with connectomic and delay data described in Section **Structural connectivity and path-length data**) that are not accessible in the linear regime. These are highly likely to be important in supporting the wide variety of functional states that underpin higher brain function.

**Fig 5:**
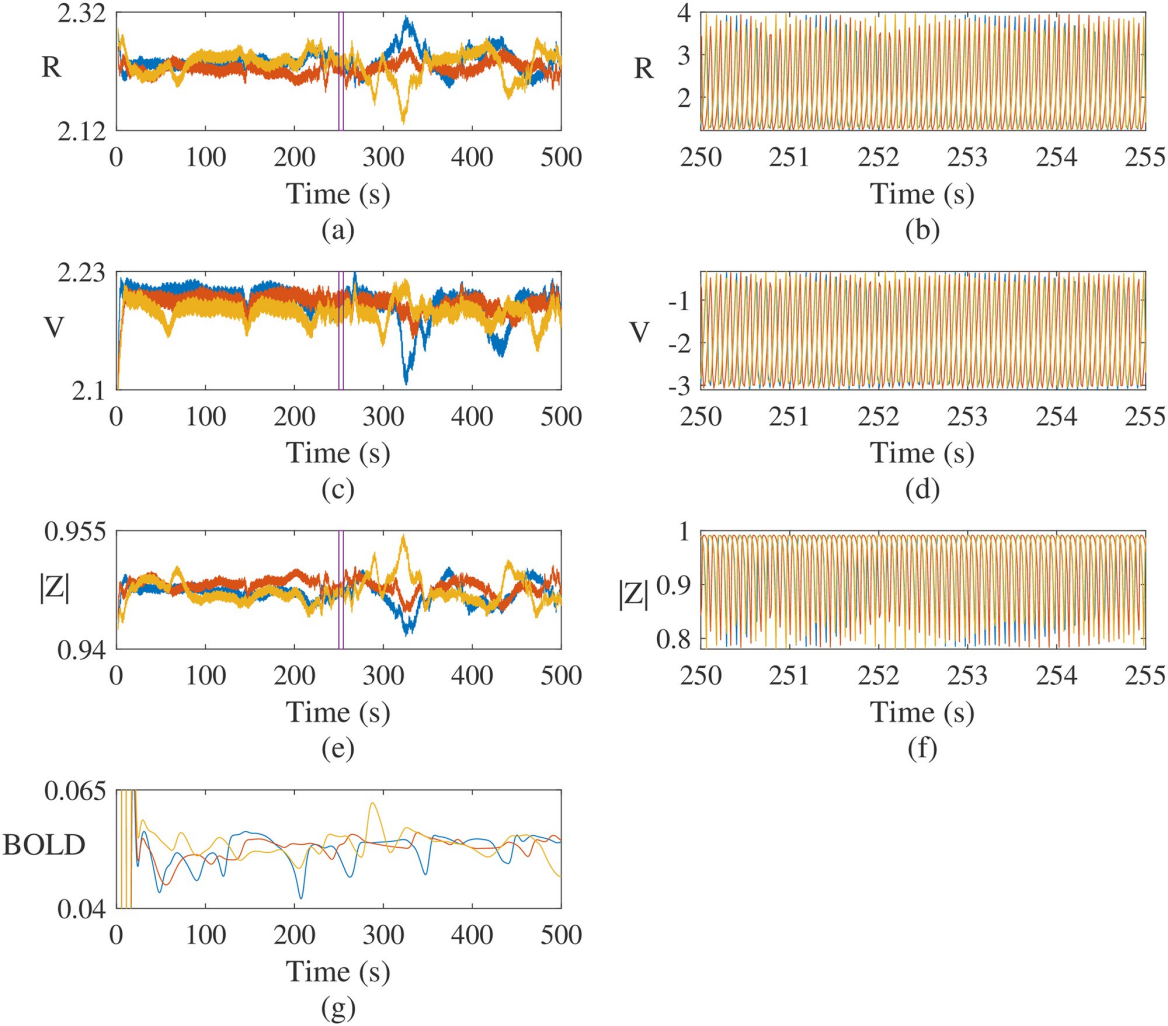
Example timeseries for local excitatory population variables, *V*_*E*_, *R*_*E*_ and synchrony |*Z*_*E*_| obtained via direct simulation of the network model (7)–(10), employing connectomic and delay data described in Section **Structural connectivity and path-length data**. Note only results from 3 selected nodes are shown, for clarity. The left pericalcarine cortex [node 20] (blue), left supramarginal gyrus [node 30] (red) and right fusiform gyrus [node 40] (yellow). (a), (c) and (e) show the amplitude envelope of the whole timeseries for each variable, given by the absolute value of the Hilbert-transformed signal. Within the time intervals indicated by the inset purple boxes, (b), (d) and (f) show a sample of the raw timeseries. Panel (g) shows a synthetic BOLD signal, computed via (17) from the *R*_*E*_(*t*) timeseries.

Fig 5 provides exemplar time series of activity (*R*(*t*) and *V*(*t*)) and underlying spiking coherence of the population (|*Z*(*t*)|) from selected nodes in the network, obtained from direct simulations. These highlight both the complex oscillatory patterns generated over short timescales (b, d, f), and the longer timescale variation in waveform amplitude (a, c, e) that the network supports. This activity stands in contrast to simpler neural mass models, such as the popular Wilson–Cowan model in which behaviour is largely limited to sinusoidal-type oscillations, or other more complex examples such as Jansen–Rit for which notions of within-population coherence are not available, and for each of which features of key biological and neurophysiological relevance such as gap junction coupling are not accommodated. From the perspective of “intrinsic coupling modes”, MEG is used in this manuscript to assess FC by maximization of imaginary coherency on fast time scales, whereas correlations between BOLD responses attributed to relatively slower time scales should be understood as envelope correlations, see, e.g., [72]. Given this, there ought to be, and typically there is, some relation between these two modalities, e.g., when considering the envelope of MEG time series as shown in Fig 5(g), which shows a synthetic BOLD signal computed via (17) from the *R*_*E*_(*t*) timeseries.

**Fig 6:**
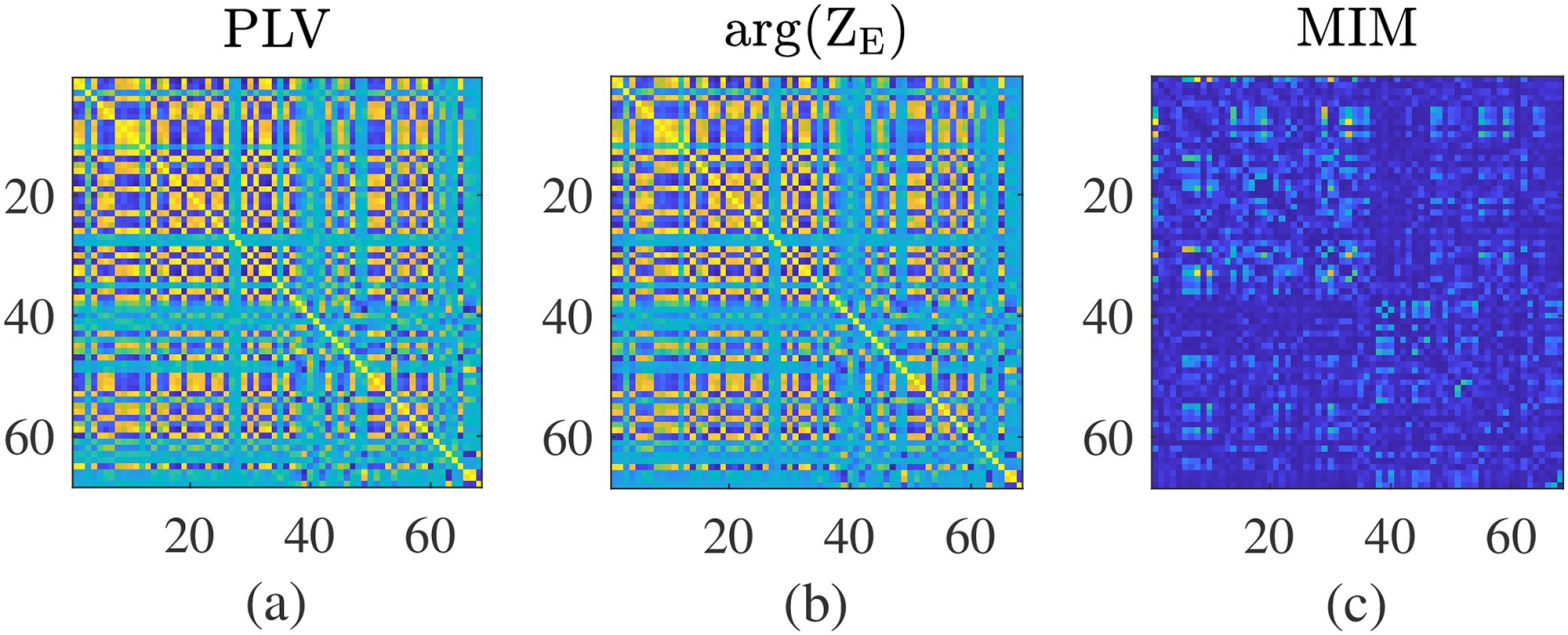
Comparsion of methods to compute functional connectivity panel (a) shows the PLV matrix computed from the simulated MEG signal using the Hilbert transform to the phase 16, panel (b) shows the corresponding PLV using the mean phase of each population given by arg(*Z*_*E*_) and panel (c) shows the FC from the simulated MEG signal using the MIM method described in section **Linear stability analysis**.

In Fig 6 we show that by using the PLV measure the phase coherence between the mean phase of each population given by arg(*Z*) is closely matched by the PLV derived from the corresponding simulated MEG signal. These show qualitative differences to the network derived using the MIM measure. However, the use of MIM might be considered a more appropriate measure of FC as it has been developed as a real-world signal processing tool for MEG data. Thus despite the computational simplicity and ease of constructing static PLV measures of FC within the modelling framework we would advocate for MIM instead.

**Fig 7:**
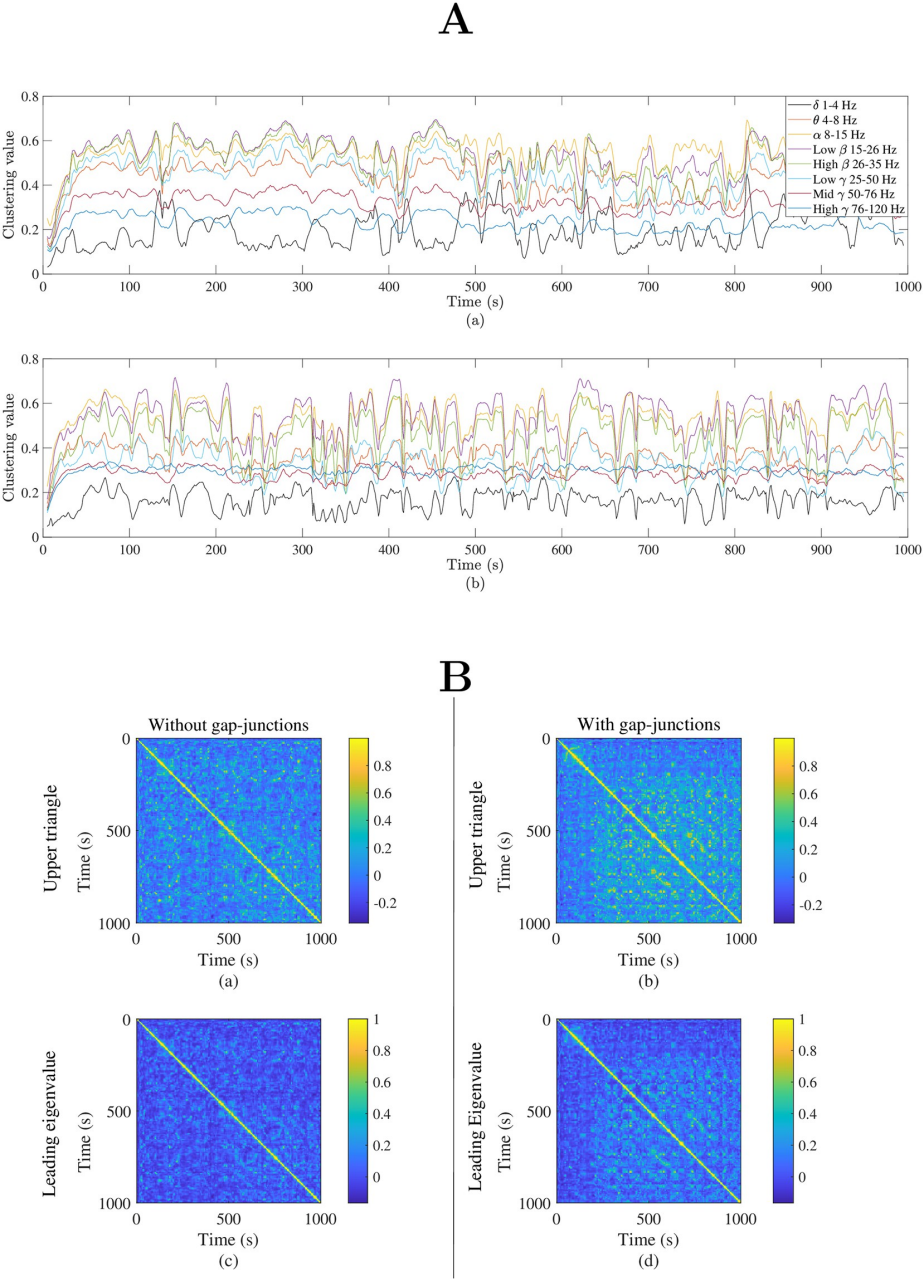
Gap junction coupling supports rich and dynamic neural activity. Direct simulations of the network model (7)–(10) (together with connectomic and delay data described in Section **Structural connectivity and path-length data**) provide simulated data in the absence ($\kappa_{v}^{ab}=0$) and presence ($\kappa_{v}^{EE}=0.01$ and $\kappa_{v}^{II}=0.025$) of gap junction coupling; dynamic FC (dFC) matrices are obtained by employing the *R*_*E*_ component of node activity. **A** Network averaged structure-function clustering coefficient $C_{\text{wsf}}$ (19) computed via simulated MEG dFC (see Section **Functional Connectivity**) for each of the listed frequency bands using a sliding time window of width 10 s and 90% overlap. **B** Following [75], the instantaneous phase of synthetic BOLD signals (17) is computed with the Hilbert transform and used to compute dFC matrices whose entries comprise the cosine of the pairwise phase differences. To interrogate their time-variation, the leading eigenvector (that corresponding to the largest eigenvalue) and the vector of upper triangular values is extracted and time-correlation assessed via Pearson correlation.

The key influence of gap junction coupling on emergent network behaviour across the various frequency bands of importance in the analysis of neuroimaging data is made evident in Fig 7 in which we employ a selection of metrics to analyse the dynamical features supported by the model in the presence and absence of such coupling. These aspects are of especial importance in the context of recapitulating the dynamical repertoire observed in functional patterns both in the context of task-switching [73] and fluctuations in resting state [74] and are not captured by the averaged (static) FC patterns considered above. Specifically, we compute dynamic FC (dFC) matrices from the *R*_*E*_ component of simulated data in two different ways. First, simulated MEG dFC (obtained as described in Section **Functional connectivity**) is computed for various frequency bands over sliding windows of width 10 seconds with a 9 second overlap. The resulting FC patterns are interrogated via the network-averaged structure-function clustering coefficient (19), providing a convenient metric to visualise the influence of anatomical structure on evoked activity patterns and how congruence between structure and function (or its absence) evolves over time. This is presented in Fig 7A. Secondly, we follow [75] and employ synthetic BOLD signals via (17). The instantaneous phase of these signals is computed via Hilbert transform, and the cosine of pairwise phase differences between cortical areas provides a dynamic FC matrix for each time point. To analyse these patterns, the leading eigenvector and the vector of upper triangular values are extracted from these matrices, and their autocorrelation computed via the Pearson metric, as presented in Fig 7B.

We see that the divergence between connectivity structure and evoked function, as measured by the network averaged structure-function clustering coefficient $C_{\text{wsf}}$ differs significantly between frequency bands. Moreover, while small fluctuations are evident in each frequency band in panel A(a), in the presence of gap junctions, intermittent periods of strong SC-FC disparity are induced in all frequency bands (panel A(b)). Similarly, Fig 7Ba and 7Bb highlight that a rather richer correlation structure in the time-evolution of dFC is supported by gap junction coupling, compared to that obtained in its absence.

S1 and S2 Videos provide a further exposition of the influence of gap junctions on network behaviour. Here, we project the local structure-function clustering coefficient *C*_wsf_(*i*) that arises in the network in the presence and absence of gap junction coupling (computed via simulated MEG dFC and filtering in the *α* band; see Section **Functional Connectivity**) onto a reference cortical surface. Cortical surface visualisations in the videos were made using BrainNet Viewer [76].

**Fig 8:**
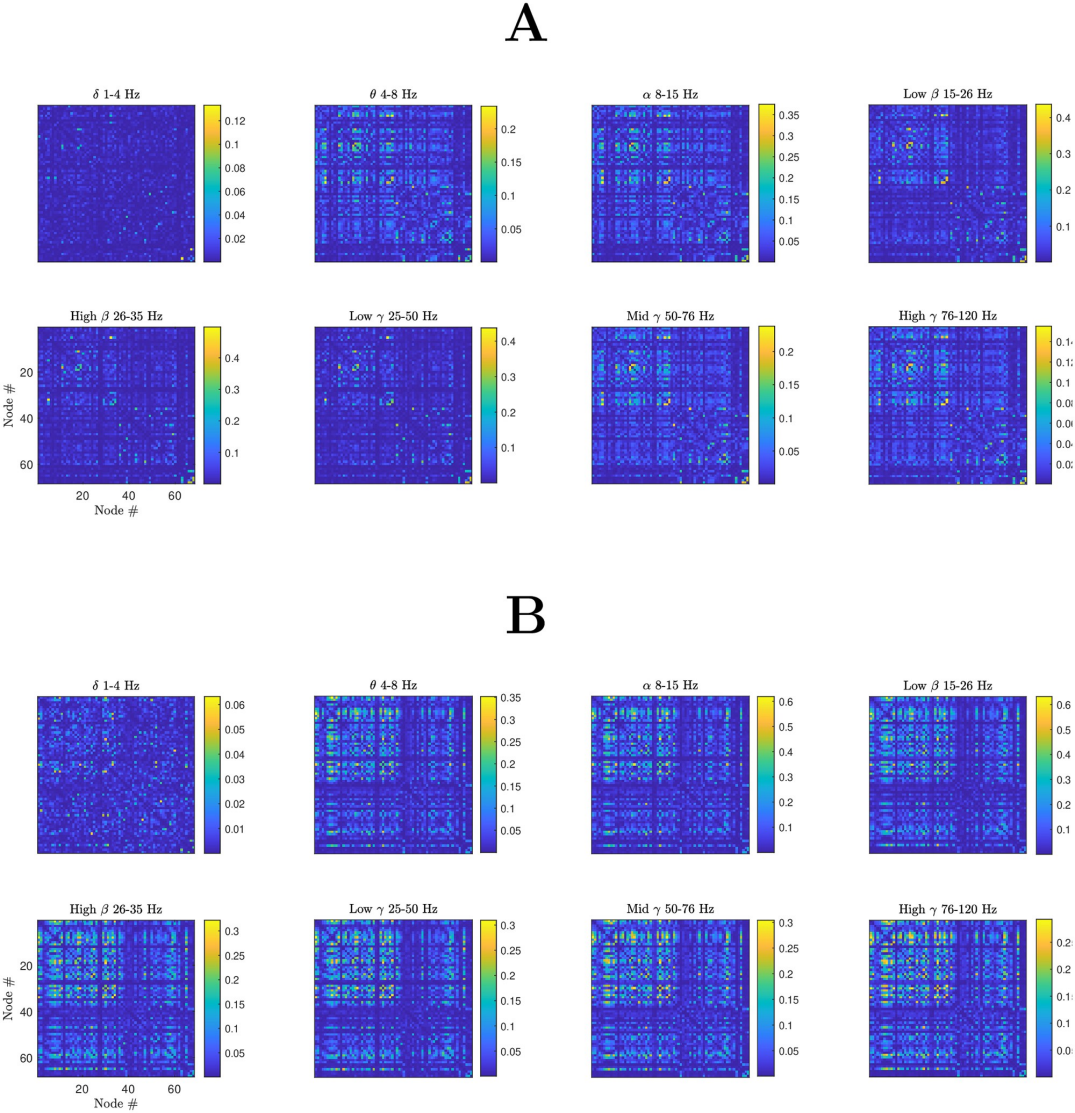
The importance of non-linearities in the system in generating simulated frequency-band filtered MEG FC more reminiscent of empirical data. MEG FC obtained as described in Section **Functional connectivity** for parameter values in which the system is (a) poised in the neighbourhood of a Hopf bifurcation (*k*_ext_ = 0.2); (b) in the nonlinear regime (*k*_ext_ = 0.5) in which larger oscillations and more complex dynamics are obtained, supporting a range FC patterns across frequency bands.

Fig 8 considers frequency band-filtered FC patterns obtained from the model in more detail. Here, simulated MEG FC is computed for the entire timeseries (as described in Section **Functional connectivity**) highlighting that a rich diversity of FC patterns across bands, as observed empirically, is not available in the linear regime (namely the regime close to a bifurcation, where one typically expects a linearisation to be good approximation of the full dynamics, at least for a supercritical bifurcation). In this regime, the linear analysis presented earlier applies, and a reasonable prediction of FC is available. However, the FC patterns presented in panel (a), for which the model is poised in a neighbourhood of a Hopf bifurcation which gives rise to network oscillations, show relatively little variation across frequency bands as would be expected empirically and so this predictive power is arguably of limited utility. In contrast, when nonlinearities play a dominant role (panel (b)), in which variation increased of global coupling strength places the model in a regime of larger oscillations and more complex network dynamics, stronger variation in FC is observed across frequency bands.

**Fig 9:**
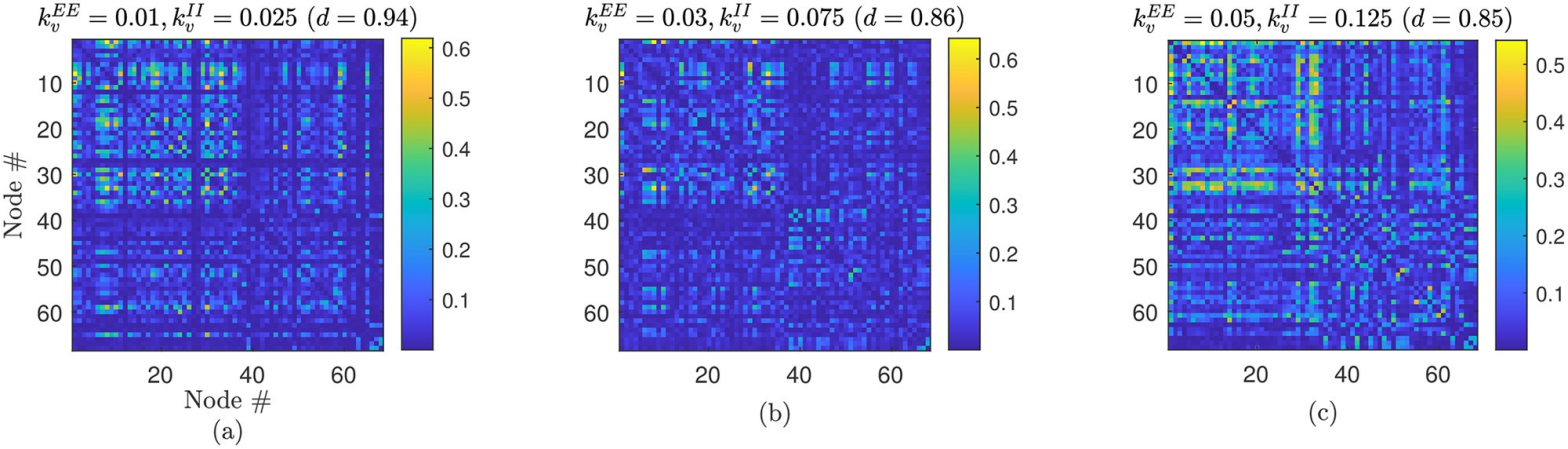
Gap junction coupling facilitates improved fits to empirical data. Panels (a–c) present simulated *α* band MEG FC, and its similarity to empirical resting-state data (see Section **Functional connectivity**) for three different values of gap junction coupling strength. Similarity to empirical FC is measured by the Pearson distance *d*.

Correspondence between simulated and empirical data is further considered in Fig 9. Here, we return to the importance of gap junction coupling on the model dynamics and present exemplar results highlighting that inclusion of such coupling facilitates improved fits to resting-state MEG FC (see Section **Functional connectivity**) in the *α* band. Distinct differences in FC are observed in simulated FC as gap junction strength is increased between panels (a–c) further underscoring its importance in mediating FC patterns. Moreover, the observed goodness-of-fit to empirical data first improves and then worsens as coupling strength is increased. This local minimum is identified with only small manual increase in $k_{v}^{EE}$ and $k_{v}^{II}$, suggesting that these parameters provide a natural choice for more comprehensive future fitting studies leveraging recent advances in parameter optimisation for whole brain models based on covariance matrix adaptation evolution strategies and Bayesian optimization [77].

**Fig 10:**
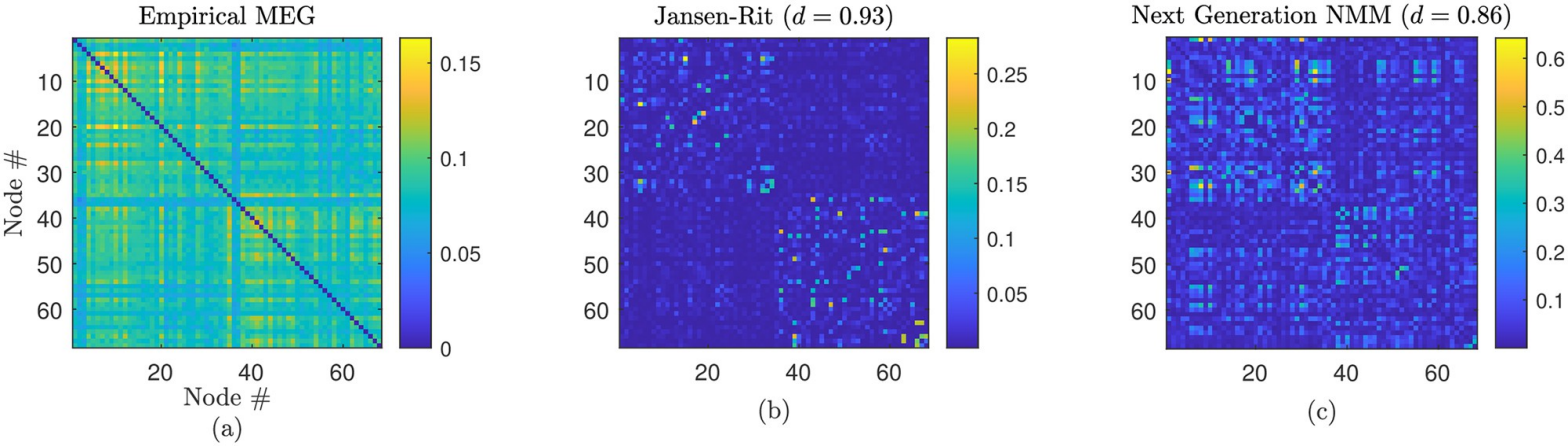
Comparison of empirical MEG FC (a) and simulated FC obtained from (b) the Jansen-Rit model [16], (c) the next generation neural mass model. Panel (c) is reproduced from Fig 9(b); parameter values in the Jansen–Rit model are taken from [35]. Similarity to empirical FC is measured by the Pearson distance *d*.

Lastly, Fig 10 brings together some of the ideas discussed above to compare the performance of our next generation neural mass model in fitting to empirical MEG FC (as shown in Fig 9(b)) and that of the phenomenological model of Jansen and Rit [16]. For the latter, the parameter values are chosen to be consistent with [35]. This simple example serves to illustrate how the more complex dynamics generated by this new modelling framework supports the generation of patterns of brain network activity more reminiscent of MEG data in the sense that a lower value of the Pearson distance between empirical and simulated FC is readily obtained.

## Discussion

The question as to how large-scale spatio-temporal patterns of brain activity, of the type that can be readily imaged using modern neuroimaging modalities such as EEG/MEG and fMRI, emerge from underlying neuronal interactions is one that continues to generate much research activity in both experimental and theoretical communities. In this paper we have pursued a mathematical and computational approach, building on recent advances in mean field modelling of neural population activity, to examine mechanisms for shaping whole brain functional dynamics at rest. We are certainly not the first to work in this space, and the use of neural mass models for this purpose has a long history as exemplified by the activity of *The Virtual Brain*, a neuroinformatics and simulation platform, which allows connectome-based whole-brain modelling [78]. Indeed a recent use of this platform, with a model similar in spirit to the one presented here, although restricted to instantaneous current synapses (and also lacking reversal potentials) and without electrical gap junction coupling, has been presented in [79] for mouse connectome data. Interestingly, even without some of the physiological realism and human connectome data that we have incorporated here, the authors were able to link the fast temporal microscopic neuronal scale to the slow emergent whole-brain dynamics and show that cascades of neuronal activations spontaneously propagate in resting state-like conditions. The work presented here is complementary to this and further shows that the local modulation of gap junction strength provides another mechanism by which physiology at the small scale can affect brain dynamics at the large scale. Without the need for receptors to recognise chemical messengers, gap junctions are much faster than chemical synapses at relaying signals. The synaptic delay for a chemical synapse is typically in the range 1 − 100 ms, while the synaptic delay for an electrical synapse may be only about 0.2 ms. Little is known about the functional aspects of gap junctions, but they are thought to be involved in the synchronisation of neurons [80, 81] and contribute to both normal [82] and abnormal physiological brain rhythms, including epilepsy [83]. Moreover, it has recently been hypothesised that activity-dependent gap junction plasticity can act as a mechanism for regulating oscillations in the cortex [84].

In the modelling study presented here, we have found that the incorporation of gap junction currents facilitates the generation of rich and complex neural activity time-series and corresponding functional connectivity patterns, and improved fits to resting state data. Given that these are so often neglected in phenomenological neural mass models this is yet further reason for the use of the more principled next generation mass model utilised here in future large scale brain modelling. This would seem to be especially important given that gap junctions are ubiquitous throughout the human brain, being found, for example, in the cortex [85], hippocampus [86], the inferior olivary nucleus in the brain stem [87], the spinal cord [88], and the thalamus [89].

## Future work

Although, for ease of exposition, we have focused on a homogeneous large scale brain model (in the sense that all nodes in the network are identical), this is easily relaxed in the computational framework we have implemented here. In future work we plan to explore this further, and fit a more heterogeneous model against existing multimodal (fMRI-EEG) imaging datasets for simple sensory tasks [90–92]. This data exhibits a more dynamic component (including negative BOLD) than found in resting state data, with post-stimulus responses of the type that the next generation neural mass model (without gap junction currents) has had previous success in reproducing for MEG data showing post-movement beta rebound [24]. Such a fitting and optimisation task is no small challenge, though the relatively new history-matching approach (for exploring parameter space and identifying the parameter sets that may give rise to acceptable matches between the model output and the empirical data) that has had strong success in other areas of science and engineering [93] provides a powerful potential approach, as does multiple objective optimisation that respects physiological constraints [94], and/or the use of techniques from data assimilation [95] and Bayesian inference [96]. Moreover, when combined with a recent Bayesian model comparison framework [97], this may readily allow for the selection of plausible hypotheses about the function of gap-junction coupling. The construction of ever more realistic cortical network models allows for the possibility of practical *in silico* sandboxes for developing, say, new protocols for transcranial magnetic stimulation (via its effects on emergent FC patterns) as piloted in [29], or developing further insight into mechanisms of distributed working memory as in [98] (that made use of phenomenological neural mass rate models). The modelling framework and the suite of C++ tools in NFESOLVE that we have presented here will allow us and others to take this programme forward, as well as to incorporate other important dynamic components such as adaptation [99, 100] and plasticity [101, 102].

## Supporting information

S1 File NFESOLVE: An object-oriented differential equation solver. This Supplementary file describes the implementation and functionality of NFESOLVE. This is a purpose-built suite of numerical solvers implemented in C++ for simulating neural mass and field problems.
(PDF)

S1 Video Video showing the simulated dynamics of the next generation neural mass model without the inclusion of gap-junction coupling, projected onto a reference cortical surface. Coloured according to the value of the local structure-function clustering coefficient, with warmer colours indicating higher values.
(AVI)

S2 Video Video showing the simulated dynamics of the next generation neural mass model with the inclusion of gap-junction coupling, projected onto a reference cortical surface. Coloured according to the value of the local structure-function clustering coefficient, with warmer colours indicating higher values.
(AVI)
