# Supplementary material for: Whole brain functional connectivity: Insights from next generation neural mass modelling incorporating electrical synapses
Source: PLoS Comput Biol. 2024 Dec 5;20(12):e1012647. doi: 10.1371/journal.pcbi.1012647 (PMC11651611; doi:10.1371/journal.pcbi.1012647)
Supplement: S1 File — This Supplementary file describes the implementation and functionality of NFESOLVE. This is a purpose-built suite of numerical solvers implemented in C++ for simulating neural mass and field problems. (PDF) [file pcbi.1012647.s001.pdf]

NFESOLVE: An object-oriented differential equation solver

Michael Forrester[1], Sammy Petros[1], Oliver Cattell[1] Yi Ming Lai[2], Reuben D O'Dea[1], Stamatios Sotiropoulos[2], Stephen Coombes[1*].
1 Centre for Mathematical Medicine and Biology, School of Mathematical Sciences, University of Nottingham, Nottingham, NG7 2RD, UK.
2 Faculty of Medicine & Health Sciences, University of Nottingham, Nottingham, NG7 2RD, UK.
* stephen.coombes@nottingham.ac.uk

Abstract

This Supplementary file describes the implementation and functionality of NFESOLVE. This is a purpose-built suite of numerical solvers implemented in C++ for simulating neural mass and field problems. NFESOLVE takes advantage of parallel processing (via the openMP package) together with efficient, sparse data-structure memory storage, and adaptive time-stepping that allows for delay differential equation problems to be integrated in an efficient manner. A key part of the efficiency of this suite is in the storage of only those delayed variables that are required (in contrast, most pre-existing solvers require delay states to be computed for the entire system) and in computing required delayed states that fall between time-steps of past solution states via a third order Hermite interpolant. Due to the adaptive time-steps, the size of the history array is dynamic, only ever storing the values necessary to compute the next step in the integration. The code, along with a selection of example problems, is stored on a GitHub repository and is available to download at `https://github.com/UoN-Math-Neuro/NFESOLVE`. The example files include functions and parameters describing a neural mass model, which may be edited to suit the particular model of interest to the user. For ease of use, these files use the Armadillo linear algebra library which has a functionality similar to Matlab.

1 Introduction

NFESOLVE is a suite of numerical solvers implemented in C++ to aid in the solution of non-local and nonlinear neural mass and field equations with multiple delays. The chosen standard selected here is C++11 [1]. The Armadillo library [2–4] is used for its efficient vector and matrix classes. This uses BLAS (Basic Linear Algebra Subprograms) [5] and LAPACK (Linear Algebra Package) [6] for linear algebra, and can automatically use OpenMP [7] parallelisation to speed up intensive computations. Additionally, sparse matrix structures are supported. Here, we employ the OpenBLAS [8] implementation for the back-end as it is a highly optimised version of BLAS.

The entire suite of code, along with a selection of example problems, is stored on a GitHub repository and is available to download at `https://github.com/UoN-Math-Neuro/NFESOLVE`.

In the following, we provide explanations of the classes that make up the software, split into four main groups: mesh handler, ordinary differential equation (ODE) solvers, delay differential equation (DDE) solvers, and sparse DDE solvers.

2 Mesh handler design

As part of the NFESOLVE package, a mesh handler toolkit is included. This provides a base class to store details of a geometric mesh, along with subclasses for specific mesh types, and a quadrature library to generate the weights and points associated with various quadrature rules depending on the element type and dimension of the geometry. Although these classes can be applied in many different settings and to a wide selection of problems, the main motivation behind developing them as part of the NFESOLVE library was to manage the spatial sums and integrals that are typically part of neural mass and field models.

2.1 Mesh class and subclasses

The *Mesh* class is a base class designed to store the coordinates and element data that make up a finite element mesh. It is built to handle non-hybrid polygonal meshes in any given dimension. The majority of the methods in this class are 'getter' methods to return the attributes that the class holds. These include custom methods for returning specific individual element connectivity arrays and grid point coordinates. If the user at any point wants to change the grid points or connectivity data without instantiating a new object, the class provides 'setter' methods to do so. Finally, there is an output method that allows the mesh data to be written to a file so that, if the user requires, the mesh can be utilised outside of the scope of the NFESOLVE library. A custom data file structure to facilitate the writing out of this data has been designed.

Mesh Data File Format

```
DIMENSION OF MESH
NUMBER OF GRID POINTS
NUMBER OF ELEMENT VERTICES
NUMBER OF ELEMENTS

LIST OF GRID POINT COORDINATES

LIST OF ELEMENT CONNECTIVITY ARRAYS
```

This format is also required by the *MeshFromFile* subclass in order to read in meshes from external sources. Users are expected to formulate their mesh data into a data file beforehand, assuring that it is structured in this specific format.

For basic meshes in lower dimensions, the user may not have a data file readily available and would be required to generate this file before they could proceed. In order to provide a good user experience, we have supplied a selection of other subclasses to the *Mesh* class which can be used to automatically generate standard meshes for simple domains, such as intervals and rectangles, which are the shapes of choice for many idealised studies.

The first of these, namely *Regular1DGrid*, has two possible constructors. The first generates a linearly spaced 1D grid in a similar way to the `linspace()` function found in languages such as MATLAB and Python. Users supply a start and end point along with the number of points they would like the grid to have. The grid points and element connectivity data are then generated automatically. Alternatively, the user can supply a pre-constructed vector of grid points (without regard to whether they are uniformly spaced) and the constructor will build the element connectivity array for those given grid points.

Two other standard classes that are provided are *Regular2DTriGrid* and *Regular2DQuadGrid*. These provide discretisations of rectangular domains, using triangular and quadrilateral elements respectively. They each have three possible constructors but these have the same arguments for both element types. Similarly to the *Regular1DGrid* class, the first two of these allows the user to specify the start and

end points for both vertical and horizontal directions. There is then the option to input either one or two values dictating the number of grid points in each direction. If only one value is entered then there will be the same number of grid points in each direction. Using these inputs, a rectangular lattice of grid points is formulated and the element connectivity array is generated based on which element type is required. The third type of constructor takes in an array of pre-defined grid points for each direction and then builds the rectangular lattice and element connectivity array from the supplied points. For the quadrilateral grid, each grid point is connected to its neighbouring grid points, thereby creating rectangular elements. The triangulated grid is similar to the quadrilateral grid, however, each rectangle is divided diagonally in half from bottom right to top left, thus generating triangular elements.

2.2 *QuadratureLibrary* namespace

Another key part of the NFESOLVE library, that goes hand in hand with the mesh classes, is a suite of quadrature rules that allow for numerical integration to be performed over a mesh. This is particularly relevant to neural field models due to the spatial integral term that is typically present. This is designed in the form of a namespace instead of a class. A namespace in C++ is the equivalent of a class with static methods. These are methods that do not require an object of the class to be instantiated before being used.

The *QuadratureLibrary* namespace contains methods for generating the quadrature weights associated with interval, triangular and regular quadrilateral element types. These methods all take in an object of type *Mesh*, which contains the relevant information about the element type and dimension, allowing for the appropriate rule to be applied. The simplest of the rules in the suite are those that only utilise the vertices of the elements. These rules do not require any additional quadrature nodes to be generated, and for higher dimensional meshes correspond to the trapezoidal rule in one dimension. They can therefore be applied directly to the original meshes and can be very useful in providing an efficient approximation to an integral where integrand values are only known at the supplied mesh points, such as when using real-world data. For interval domains, a method to generate the weights for Simpsons's rule has also been implemented.

3 ODE solver design

Included in the codebase are a series of classes that are designed to facilitate the numerical solution of ODEs. For flexibility, a polymorphic design structure is used. This relies on a base interface class, named *ODEInterface*, which provides a structure for defining a specific problem to be solved. This allows for the solver classes to accept any ODE problem that is built from this interface. We make use of an abstract class, named *AbstractDESolver*, to handle essential features, and deriving from this, we employ a *AbstractODESolver* class to act as a base for different numerical ODE solvers. This contains the properties that are unique to just ODE solvers. The specific solution algorithms that have been implemented as part of the NFESOLVE library include both a 3rd and 4th order fixed-step Runge-Kutta scheme, along with an adaptive-step 3(2) order Runge-Kutta scheme.

3.1 *ODEInterface* Class

In order to solve a given ODE, a solver class needs to be able to accept an object that contains all the relevant information pertaining to the desired problem. A base interface

class, named *ODEInterface*, contains common attributes that all ODE problems share (such as a 'right-hand-side' or vector field 'F'). As this is an interface, it is never directly instantiated, but it does allow for a solver class to expect an object that follows a certain contract and for it to call any relevant methods. Users are able to add in additional information or processing steps into their derived class, meaning key components such as model parameters or extra function evaluations can be incorporated. The only requirement is that they implement any pure virtual methods defined in the *ODEInterface* class. A pure virtual method is a method that has no defined implementation and must be overridden by any derived class that is itself not abstract.

To illustrate the design of this interface, we use the typical form of an ODE, given by

$$y'(t) = F(t, y(t)), \qquad y(t_0) = y_0. \tag{1}$$

For simplicity, we refer to the variable t as representing 'time'. The *ODEInterface* class contains a pure virtual method for computing the right-hand-side, named `ComputeF()`, which is built to take in a time, t, and a state vector, y, as input arguments. This is pure virtual as there is no generic implementation; it must always be defined in a derived class pertaining to the specific problem. There also exists a virtual method for computing the analytic solution at a given time point, if the analytic solution is known. This typically will not be used, hence why it is just a virtual method and not a pure virtual method, however it is included so that it can be used if required. An example where this method would be useful is for processes such as numerical error analyses. It is especially important for the user to make sure that the contract for these methods always remains consistent across all derived classes that they create, as the solvers which utilise the interface are only aware of the methods and variables that are defined in the interface, not any other methods or variables which the user may implement within their child classes.

3.2 *AbstractDESolver* class

Similarly to the *ODEInterface* design, we wish to employ an inheritance-based architecture for the suite of differential equation solvers. Before moving directly to discussing the ODE solvers, it is pertinent to discuss the commonalities that arise across numerical solvers for all forms of differential equation (ODEs, DDEs, etc.). Although the numerical algorithms for each type of differential equation can vary in a multitude of ways specific to the type of problem that is being solved, there are a number of common properties that are shared amongst all types of solvers which can be abstracted out into a parent class that acts as a base for all types of numerical differential equation solver.

Firstly, no matter which type of equation is under scrutiny, there will also be an initial and final time point for which the solver is instructed to compute the solution between. Another key aspect that is shared across all numerical differential equation solvers is the current step size for which to integrate forward with. Whether this has a fixed value or it adapts according to the solution, it is not distinct to any type of problem and can therefore be defined outside of any specific solver classes. The main piece of information that is required by any numerical solver is the current solution state of the system. The parent class, *AbstractDESolver* holds these attributes. We also opt to include a number of variables for facilitating the output of the current solution state so that the user can access it externally to the application. These include the name of the output file that the solution will be saved to, an array containing the indices of the solution state that are to be saved (in case the user does not wish to unnecessarily store data that is not of interest to them), a 'save gap' variable which determines how frequently the stepped solution will be saved to the output file, and a 'print gap' variable to specify the frequency at which the solution should be printed to the console (if the user wishes to visualise the numerical data in real time).

In terms of the methods that are available in this abstract class, the main one is a pure virtual method named `Solve()`. In all of the derived solver classes, this is where the specific solution algorithms will be implemented. We choose to declare this method in this parent class to keep the method name reserved for implementation. As is standard, 'setter' and 'getter' methods are provided for all the variables listed, allowing for multiple different simulations to be run back-to-back from the same solver object. Additionally, this parent class contains two non-virtual methods which format and then print a given solution state to the console or save it to a data file, while respecting the previously discussed 'print gap' and 'save gap' variables. Finally, a general method to print an elapsed time frame to the console is also included. This can, for example, be used within the child solver classes to display the time taken for the `Solve()` method to complete.

3.3 *AbstractODESolver* class

Although the *AbstractDESolver* class handles the key properties that are shared across all numerical differential equation solvers, it does not account for anything specific to ODEs. Following a similar approach to the *AbstractDESolver* design, a new abstract class that inherits from this parent class is provided to cater for ODE problems. This class is called *AbstractODESolver*. As it inherits from *AbstractDESolver*, all the variables and methods discussed in Sec. 3.2 are available to access. The main distinguishing feature that gears this class specifically towards ODE problems is that it stores a pointer of type *ODEInterface* that will be used in a polymorphic way to access the specific ODE problem that is being solved. If the user wishes at any point to change the ODE which is being pointed to but maintain the solver parameters defined within a solver object, a setter method has been provided as part of this class. Any classes which derive from *AbstractODESolver* are now fully equipped with access to everything they need to implement a numerical ODE solution algorithm. This design structure means that new solvers can be added in to the NFESOLVE library.

3.4 *RungeKuttaSolver* classes

One of the most widely used algorithms for numerically solving differential equations is the Runge-Kutta scheme. Provided with the NFESOLVE package are a selection of Runge-Kutta solvers for ODEs. In order to facilitate generating Runge-Kutta classes of any specific order, a key design choice is to implement a base class, named *RungeKuttaSolver*, which derives from *AbstractODESolver* and contains methods for both the non-adaptive and adaptive Runge-Kutta algorithms. These methods take in the coefficients as input arguments, allowing for any Runge-Kutta scheme to be utilised. Before the actual implementation of the algorithm, the methods begin by printing a header to the console which details all the information about the chosen solver and the chosen parameters. A clock is started to monitor the elapsed wall time (real-world time taken to complete a task) for the duration of the stepping process. At the end of each computed step, a check is made to determine if the step count is divisible by either the print gap or save gap variable. If the check returns true then the corresponding output method (defined in *AbstractDESolver*) is called. The stepping algorithm proceeds to the next step and this process is repeated until the time variable reaches the final time that was specified by the user. Finally, the total elapsed wall time is printed to the screen. As these algorithms are only implemented once, instead of repeatedly for each individual Runge-Kutta solver, this allows for any future changes to apply globally to all specific Runge-Kutta solvers. To implement a specific Runge-Kutta method, we simply create a new class that derives from the *RungeKuttaSolver* class and override the

pure virtual `Solve()` method (defined in *AbstractDESolver*) to call either the non-adaptive or adaptive Runge-Kutta algorithm.

Thus far, there are two fixed-step explicit Runge-Kutta solvers and one adaptive-step explicit Runge-Kutta solver that have been developed and tested. The first of the three implemented solvers is a 3rd order method, known as Kutta's method. This is defined in a class named *RungeKutta3Solver*. Internally, pre-defined private variables contain the relevant coefficients for this method. When an object of type *RungeKutta3Solver* is instantiated, the constructor requires the user to supply a reference to a child object of *ODEInterface*, an initial condition, the initial and final times that the solution is required between, the step size they wish to be used, a name for an output file, a 'save gap', and a 'print gap'. Optionally, the user may also supply a vector containing a list of indices of the state variables they wish to be outputted. If this is not specified, the default implementation is to output every element of the state vector. To begin the stepping process, the user simply calls the `Solve()` method.

The second of the fixed-step solvers included in the NFESOLVE library is a 4th order method, defined by the classical Runge-Kutta formula. This class is named *RungeKutta4Solver* and is implemented analogously to the *RungeKutta3Solver* class.

The third and final ODE solver that has been implemented to date is an adaptive-step scheme that uses the 3rd order Runge-Kutta method detailed above for the main algorithm. The step size adaptation is determined by using this approximation in conjunction with an embedded 2nd order Runge-Kutta method whose coefficients are computed based on the 3rd order coefficients. This approach is very similar to that used in MATLAB's ode23 solver [9]. We name this adaptive solver class *RungeKutta32Solver* as it is a 3(2) scheme. Instead of the constructor requiring the user to pass in a step size as an argument, they instead input their choice of the absolute and relative tolerances that comprise the adaptation error condition.

4 DDE solver design

The construction and implementation of a suite of classes to solve DDEs (with constant delays) follows on from the ODE solution package by building upon and expanding the modular framework used. Developed alongside the standard DDE solvers are a set of solvers that make use of a sparse data structure to allow for certain DDE problems to be solved in an efficient manner. To facilitate the development of these solvers, three additional classes are required. The first two of these provide a storage buffer for the solution history as it is stepped forward. One of these is static (size is fixed) and is utilised by the fixed-step solvers, while the other is dynamic (size can adapt) and is employed by the adaptive-step solvers. For the sparse solvers, a custom class named *SparseDelayMatrix* implements a sparse matrix structure, based on the compressed sparse column (CSC) storage format, to efficiently store the matrix of delay states that is computed at each stage of the underlying Runge-Kutta algorithm.

4.1 *DDEInterface* class

Analogously to the ODE section of the NFESOLVE library, in order to solve a particular DDE, an interface is required to provide a base for all DDE problems to derive from. This interface is then used by the solver classes so that they may accept any DDE problem and consistently call specifically reserved methods. The class is named *DDEInterface* and it mirrors the design aspects of the *ODEInterface* class.

For a series of m distinct delays, $\tau = \{\tau_1, \ldots, \tau_m\}$, the general form of a constant delay DDE is given by

$$y'(t) = F\left(t, y(t), y(t-\tau_1), \ldots, y(t-\tau_m)\right), \quad t \geq t_0, \tag{2}$$

$$y(t) = \varphi(t), \quad t \le t_0, \tag{3}$$

where φ is a history function that defines the solution prior to the initial time, t_0. Instead of writing the right-hand-side as being a function of many delay states, it can be written succinctly as $F(t, y(t), Z(t))$, where $Z(t)$ is a matrix such that $Z(t) = [y(t-\tau_1), \ldots, y(t-\tau_m)]$. This allows for a much simpler formulation of the pure virtual method contract that is reserved to compute the right-hand-side of the problem, once again named `ComputeF()`, as only one method argument is required to encompass all the delay states. On top of the `ComputeF()` method, a new pure virtual method named `ComputeHistory()` is also required to evaluate the history function, $\varphi(t)$, when solution states that fall prior to t_0 are required. As all DDEs require a history, this method must be overridden in all child classes. Lastly, in conformity with the *ODEInterface* class, there is also a virtual method included to compute the analytic solution of the equation if it is known.

4.2 *AbstractDDESolver* class

Building upon the framework previously outlined, in order to begin the implementation of specific DDE solvers, an abstract class that contains the common properties which all DDE solvers share is defined. This class is called *AbstractDDESolver* and it derives from the general differential equation solver abstract class, *AbstractDESolver*. It is very similar to the *AbstractODESolver* class in many respects, except that instead of storing a pointer to an object of type *ODEInterface*, it now contains storage for an object of type *DDEInterface*. There are also two new additions to the set of privately stored variables that are required in order to accommodate the delays. The first of these is a vector that stores all of the delay values present in the system. This is important as the solver will need to cycle through all the delays at each time-step and compute the relevant delay state vectors so that it can populate the Z matrix. Secondly, a simple integer dictating the number of equations in the system which actually require delay states to be computed is also included. An important design choice made here was to request that the user order their system by placing the equations which require delay states to be computed at the top of the state vector. This consideration was made to prevent unnecessary delay states being computed that would never be needed, therefore saving memory and computation time.

4.3 *HistoryBuffer* classes

So as not to waste memory unnecessarily, the implementation of the ODE solver suite in the NFESOLVE library only stores the current state of the system as it is evolved. As the solution is outputted to a file (depending on the the 'save gap' the user has set), there is no need to store all the past solution states in memory, allowing resources to be reserved purely for the computation process. However, when it comes to DDEs, in order to progress the solution at any given time point, a solver must have access to the history of the solution so that it is able to compute the necessary delay states. If the stepper has not progressed past $t_0 + \tau_{\min}$, where $\tau_{\min}$ is the smallest delay value in the system, then all delay states are easily computed from the history function $\varphi(t)$. Once the stepper has moved beyond this value it will need access to the previously computed solution states in order compute the delay states needed.

Although there exist algorithms, such as the 'Natural Runge-Kutta method for DDEs', which are designed to allow the required delay states to coincide perfectly with the known solution states, in practice these schemes are typically non-viable due to the rigid restrictions imposed on the delays and solver step size. The aim of the NFESOLVE library is to remove as many restrictions as possible so that it may be used

to efficiently solve all manner of models. One way of doing this is to make use of a third order Hermite interpolant to compute delay states that fall in between the past solution states. The upside to using an interpolant is that it allows for both non-adaptive and adaptive stepping, as opposed to rigidly fixing the step size such that all delay times fall perfectly on previously computed states. The chosen Hermite interpolant [10, 11] also only requires two neighbouring solution states for it to yield a 3rd order accurate approximation of the solution.

To utilise the interpolation scheme, solution states and their derivatives will need to be stored in memory instead of being forgotten about once the stepping algorithm moves on. However, storing every single computed solution is not necessary and would quickly saturate the available memory. All that is required by the solver is to have a history of solution states stored from the current time, t_n, back to $t_n - \tau_{\max}$ (similarly to $\tau_{\min}$, the largest delay value present in the system is represented by $\tau_{\max}$). To manage this, we formulate two new classes: one for the fixed-step solvers and one for the adaptive-step solvers. The first of these is named *StaticHistoryBuffer* and, as the name suggests, does not change in size. The second is named *DynamicHistoryBuffer* and has the ability to adapt its size depending on how many entries are needed. Both of these classes follow the same structure, with the only difference being the ability to change in size.

A custom data structure is used to manage the storage of the solution states. This is made up of four components. The main three of theses are: a vector which holds the time values for which each solution state corresponds to, a matrix which holds the solution states, and a matrix that holds their respective derivatives (computed simply by evaluating the right-hand-side of the DDE). The initial length of each of these is predetermined by the chosen step size and the maximum delay value present in the system, i.e., how much buffer storage would be required to store states back in time up to and including $t_n - \tau_{\max}$. For the dynamic buffer, this initial length is estimated using a step size of 0.01, as this is the initial step size that is chosen by the adaptive-step solvers to start their stepping. If and when more storage is required, the buffer expands itself automatically by inserting in extra columns. When the buffer is full and the span covers a greater range than is needed (from t_n back to $t_n - \tau_{\max}$), then instead of adding more storage to the buffer, the earliest state is removed and replaced by the new computed state. This is where the fourth component is utilised. Instead of shifting all the states in the buffer to the left so that the earliest is always at the beginning, it is much more computationally efficient to include a single integer variable that stores the index at which the earliest state is stored, thus creating a cyclic storage system. This index is updated as and when the earliest states get overwritten. Any state can be accessed simply by using modular arithmetic with a modulus of the buffer length.

To prevent the numerous passings of states between classes, the buffer classes are designed to also contain the interpolation method required to compute a delay state for a given time point that falls within the range of values in the time buffer. This works by first finding the index in the time buffer of the nearest value to the desired delay time. Checks are made to determine whether the stored value is larger or smaller than the delay time value, thus allowing for the indices of the two neighbouring states to be found. Once the pair of column indices are determined, a Hermite interpolation algorithm is employed to compute and return the state at the required time point. If the interpolation method is called with an argument of just the delay time value then it will compute the whole delay state for every variable. There is also an overloaded method that allows for an extra argument to be included to specify distinct indices of the solution vector for which the interpolated solution is required. This is used by the sparse solvers to only compute the delay state values that are required, instead of the entire state.

On top of the methods already detailed, the classes also contain a Boolean check to

determine if the buffer is full, along with 'getters' for each individual buffer and the index of the earliest state. The constructors for both classes take in the size of the system and the initial buffer length, with the dynamic buffer also requiring the maximum delay value so that it can adapt the size of the buffer when more storage is required.

4.4 *DelayRungeKuttaSolver* classes

Building upon the *RungeKuttaSolver* class for solving ODEs, we introduce a new class, named *DelayRungeKuttaSolver*, that implements the fixed-step and adaptive-step Runge-Kutta algorithms for solving DDEs. The main stepping algorithm works analogously to its ODE counterpart. Where the delay version differs is the added computation of the delay state matrix, Z, in order to then compute the right-hand-side of the problem in question. The Z matrix is populated by looping through the delays and calling either the `ComputeHistory()` method from the given DDE problem (deriving from *DDEInterface*) or the interpolation algorithm in the history buffer, depending on whether the delay state falls before the initial time, t_0. The Z matrix has to be generated for every single stage of the Runge-Kutta algorithm, thereby adding a significant computational workload to the time-stepping process, especially when there are a large number of distinct delays in the system.

Thus far, these are the only two delay solvers that have been implemented as part of this package. Similarly to the ODE solvers, the included solvers are *DelayRungeKutta3Solver* and *DelayRungeKutta32Solver*, where the former is a fixed-step 3rd order scheme and the latter is a 3rd order adaptive-step scheme with an embedded 2nd order method for adapting the step size. There is potential for lower order methods to be included, however, the computational hardware requirements of such methods will still remain on the same order no matter what the overall order of the scheme. This means that the main trade-off will be between method accuracy and computation time. The 3rd order methods that are provided strike a healthy balance between these factors, while effectively meeting the needs of the intended use. An important point to note is that due to the interpolation algorithm requiring a known solution state either side of each delay time that a solution is required for, this presents the constraint that the step size of the time-stepper may not at any point be larger than the smallest delay in the system. If this were to happen, the latter stages of each Runge-Kutta step could cause some delay times to fall after the most recently computed state, thereby resulting in no known solution state being present ahead of the delay times. This constraint means that for systems with small delays, the step size remains restricted to a small value, thus the system could take a long time to evolve temporally.

5 Sparse DDE solver design

The DDE algorithms detailed thus far are similar to those offered by other DDE solver suites. However, for certain problems, there are computational savings that can be made to greatly reduce the number of operations carried out, thus reducing the time of computation for the solution. One such scenario would be a network model with space-dependent delays. As an example, assume that the delays in a system are defined as $\tau_{i,j} = \phi\left(||\mathbf{x}_i, \mathbf{x}_j||\right)$, where $\mathbf{x}_i$ and $\mathbf{x}_j$ are two spatial points corresponding to the ith and jth node in the system, and ϕ is some function of the distance between them. The algorithm in the standard DDE classes would populate the Z matrix column for a given delay by computing the delayed state of every single state variable at time $t - \tau_{i,j}$. However, if the right-hand-side for the ith node in the system is only dependent on the delayed state of the jth node at time $t - \tau_{i,j}$, then the Z matrix column would only

need to be populated with the jth entry instead of every single entry. In a neural network with space-dependent delays, the 'information' passing between two nodes is not dependent on the delay between any nodes other than those two. This allows for any computations of unused values in the Z matrix to be completely ignored.

5.1 *SparseDelayMatrix* class

To most efficiently reduce the memory and number of computations required, a sparse matrix data structure can be used to store the Z matrix values. The difference to conventional sparse matrices, however, is that in this case the word 'sparse' does not mean containing few non-zero elements; it instead means containing few 'used' elements. Armadillo has a built in sparse matrix structure, but as it is sparse in the conventional sense it does not allow for zero elements to be stored as values. As it is the columns of the Z matrix that are looped over when computing the delay states, Z is stored in CSC format as opposed to compressed sparse row format, due to the way the elements are stored. The constructor for this class takes in a $2 \times m$ matrix containing the index locations, in column-major ordering, of the 'used' delay state matrix values. This corresponds to the sparsity pattern of the matrix. Here m is the total number of 'used' values. It also takes in an array of length m containing the values to store in the correspondingly indexed locations, as well as the total number of rows and columns the delay matrix contains. The class also includes an overloaded parentheses operator, `()`, to access the entries of matrix under conventional matrix index notation, along with methods to return the values from specific individual columns and the corresponding row indices of each of the entries in a column.

5.2 Remaining *Sparse* classes

The remaining sparse classes follow on from their DDE counterparts. Firstly, an interface for all DDE problems backed with the sparse implementation is supplied, under the name *SparseDDEInterface*. This class contains pure virtual methods to compute the right-hand-side and history function of the DDE, utilising the sparse delay matrix structure for Z instead of a standard Armadillo matrix for passing in the delay states to the `ComputeF()` method. Similarly to the previously discussed DDE code, an abstract class to hold all the common properties of DDE solvers which uses the sparse implementation of the delay state matrix is also included. Named *AbstractSparseDDESolver*, this class derives from *AbstractDESolver* and stores a pointer to an object of type *SparseDDEInterface* for computations on the derived specific DDE problem, along with the $2 \times m$ locations matrix used for constructing the sparse delay matrix, Z. Finally, there is the suite of delay Runge-Kutta solvers that utilise the sparse implementation. A base class, deriving from *AbstractSparseDDESolver*, is included to implement the non-adaptive and adaptive generalised Runge-Kutta algorithms. This class is named *DelaySparseRungeKuttaSolver*. Within the Runge-Kutta implementation methods, the sparse delay matrix, Z, is instantiated using the locations matrix that the user will provide. At each Runge-Kutta step, when the delay times are looped over to populate the columns of Z, the methods in the *SparseDelayMatrix* class are used to return the row indices for the 'used' elements in each column. These row indices are then passed into the `ComputeDelayState()` method of the history buffer to allow it to compute only the relevant entries instead of the entire delay state, thereby greatly reducing the number of computations required.

Deriving further from this class are two specific solver classes. Similarly to the standard DDE solvers, there is a fixed-step 3rd order solver class, named *DelaySparseRungeKutta3Solver*, and an adaptive-step 3rd order solver class which uses an embedded 2nd order scheme for the step size adaptation, named

DelaySparseRungeKutta32Solver. These are implemented analogously to the standard DDE versions, except the constructors additionally take in the $2 \times m$ locations matrix dictating the sparsity pattern of delay state matrix, Z. This allows for extremely large delayed systems to be solved on lower specification machines with increased efficiency.

6 Parallelisation

Armadillo has built-in capabilities to handle parallel computations if a relevant opportunity arises, such as computationally expensive element-wise operations. It does this with the use of the OpenMP API specification for parallel programming [7]. The DDE solvers that have been developed here (both standard and sparse) all have sections where there is scope to divide up work across multiple processors in order to maximise computational efficiency. The main area where speed up is possible is within the population of the delay state matrix, Z. As this is done column by column, and all the computations are independent of one another, the work can be can be split up into chunks and distributed amongst the available processors. This is handled automatically by using an OpenMP 'for' loop with shared access to Z. When the library is compiled, it generates both a sequential and a parallel version of the code. The user then simply has to link to the parallel version and compile with the `-fopenmp` compilation flag if they wish to use the parallel capabilities of the code. Depending on the specific equation being solved, there may also be scope for the user to parallelise their overridden `ComputeF()` method.

7 Remarks

This Supplementary file gives an overview into the design, structure, and implementation of a new and bespoke suite of numerical differential equation solvers called NFESOLVE. The primary use of NFESOLVE is for the evolution of models of neural activity in both a discrete neural mass setting and a continuum neural field setting, with the incorporation of real brain data The NFESOLVE codebase is available to download in full at `https://github.com/UoN-Math-Neuro/NFESOLVE`. For further details of NFESOLVE see [12].

References

1. ISO. ISO/IEC 14882:2011 Information technology — Programming languages — C++. International Organization for Standardization, Geneva, Switzerland. 2012;27:59.

2. Sanderson C, Curtin R. Armadillo: a template-based C++ library for linear algebra. Journal of Open Source Software. 2016;1(2):26.

3. Sanderson C, Curtin R. A user-friendly hybrid sparse matrix class in C++. In: International Congress on Mathematical Software. Springer; 2018. p. 422–430.

4. Sanderson C, Curtin R. An adaptive solver for systems of linear equations. In: 2020 14th International Conference on Signal Processing and Communication Systems (ICSPCS). IEEE; 2020. p. 1–6.

5. Blackford LS, Petitet A, Pozo R, Remington K, Whaley RC, Demmel J, et al. An updated set of basic linear algebra subprograms (BLAS). ACM Transactions on Mathematical Software. 2002;28(2):135–151.

6. Anderson E, Bai Z, Bischof C, Blackford S, Demmel J, Dongarra J, et al. LAPACK Users' Guide. 3rd ed. Philadelphia, PA: Society for Industrial and Applied Mathematics; 1999.

7. Dagum L, Menon R. OpenMP: an industry standard API for shared-memory programming. IEEE computational science and engineering. 1998;5(1):46–55.

8. Xianyi Z, Qian W, Chothia Z. OpenBLAS. URL: http://xianyi github io/OpenBLAS. 2012; p. 88.

9. Bogacki P, Shampine LF. A 3 (2) pair of Runge-Kutta formulas. Applied Mathematics Letters. 1989;2(4):321–325.

10. Shampine LF. Interpolation for Runge–Kutta methods. SIAM Journal on Numerical Analysis. 1985;22(5):1014–1027.

11. Hairer E, Norsett SP, Wanner G. Solving Ordinary Differential Equations I. Springer, Berlin; 1987.

12. Petros SJ. The Numerical Solution of Neural Field Models Posed on Realistic Cortical Domains [PhD Thesis]. School of Mathematical Sciences. University of Nottingham; 2022.
